# Targeting allograft inflammatory factor 1 reprograms kidney macrophages to enhance repair

**DOI:** 10.1172/JCI185146

**Published:** 2025-01-21

**Authors:** Irma Husain, Holly Shah, Collin Z. Jordan, Naveen R. Natesh, Olivia K. Fay, Yanting Chen, Jamie R. Privratsky, Hiroki Kitai, Tomokazu Souma, Shyni Varghese, David N. Howell, Edward B. Thorp, Xunrong Luo

**Affiliations:** 1 Division of Nephrology, Department of Medicine, and; 2 Duke Transplant Center, Duke University School of Medicine, Durham, North Carolina, USA.; 3 Department of Biomedical Engineering, Duke University Pratt School of Engineering, Durham, North Carolina, USA.; 4 Department of Pathology,; 5 Department of Anaesthesiology,; 6 Department of Mechanical Engineering and Materials Science, and; 7 Department of Orthopaedic Surgery, Duke University, Durham, North Carolina, USA.; 8 Department of Pathology, Feinberg School of Medicine, Northwestern University, Chicago, Illinois, USA.

**Keywords:** Immunology, Nephrology, Chronic kidney disease, Fibrosis, Macrophages

## Abstract

The role of macrophages (MΦs) remains incompletely understood in kidney injury and repair. The plasticity of MΦs offers an opportunity to polarize them toward mediating injury resolution in both native and transplanted kidneys undergoing ischemia and/or rejection. Here, we show that infiltrating kidney MΦs augmented their own allograft inflammatory factor 1 (AIF-1) expression after injury. *Aif1* genetic deletion led to MΦ polarization toward a reparative phenotype while halting the development of kidney fibrosis. The enhanced repair was mediated by higher levels of antiinflammatory and proregenerative markers, leading to a reduction in cell death and an increase in proliferation of kidney tubular epithelial cells after ischemia followed by reperfusion injury (I/RI). Adoptive transfer of *Aif1^–/–^* MΦs into *Aif1^+/+^* mice conferred protection against I/RI. Conversely, depletion of MΦs reversed the tissue-reparative effects in *Aif1^–/–^* mice. We further demonstrated increased expression of AIF-1 in human kidney biopsies from native kidneys with acute kidney injury or chronic kidney disease, as well as in biopsies from kidney allografts undergoing acute or chronic rejection. We conclude that AIF-1 is a MΦ marker of renal inflammation, and its targeting uncouples MΦ reparative functions from profibrotic functions. Thus, therapies inhibiting AIF-1 when ischemic injury is inevitable have the potential to reduce the global burden of kidney disease.

## Abstract 2

Deletion of Allograft Inflammatory Factor-1 (*Aif1*) uniquely uncouples macrophage pro-reparative from pro-fibrotic functions, thereby promoting kidney repair while mitigating kidney fibrosis after ischemia-reperfusion injury.

## Introduction

Ischemia followed by reperfusion injury (I/RI) is a common injury to the kidney, and when compounded by maladaptive repair, it results in chronic kidney dysfunction characterized by interstitial fibrosis and tubular atrophy (IFTA) (1, 2). I/RI occurs as a result of kidney hypoperfusion in a myriad of conditions including septic or cardiogenic shock and in major surgeries such as cardiac surgery (3–5). Kidney transplantation is another scenario in which the inevitable I/RI leads to delayed graft function and poor long-term outcomes (6, 7). Therefore, I/RI creates a significant global burden of chronic kidney disease (CKD) (8) and negatively affects donor kidney utilization (9). Strategies to restrain I/RI-mediated kidney injury and improve repair would have a wide effect of decreasing the prevalence of CKD and improving transplantation outcomes.

It has been demonstrated that macrophages (MΦs) accumulate and persist in the kidney long after the initial I/RI (10, 11), resulting in a milieu that promotes MΦ alternative activation. Although these MΦs carry out reparative functions (12–14), they also promote fibrosis (15, 16). Cell-extrinsic factors controlling MΦ functional polarization have been extensively studied, but these studies have yielded limited utility for targeted therapeutics (17). In addition, MΦ-intrinsic mechanisms that control the functional polarization of MΦs are largely unknown.

Allograft inflammatory factor 1 (AIF-1) was originally identified as an IFN-γ–inducible, Ca^2+^-binding cytosolic protein (18) that is primarily expressed in MΦs (19, 20) and has since been implicated in several inflammatory diseases including autoimmune encephalitis (21), diabetes (22), rheumatoid arthritis (23), and chronic cardiac and liver allograft rejection (24, 25). However, the specific role it plays in MΦ-mediated inflammation and the therapeutic potential of its targeting remain poorly understood. Here, we showed that kidney I/RI and allograft rejection were characterized by significant upregulation of AIF-1 in kidney MΦs. Using genetic and cellular approaches, we showed that inhibition of AIF-1 expression allowed kidney MΦs to acquire proreparative functions after I/RI, thereby reducing renal tubular epithelial cell (RTEC) death, promoting RTEC proliferation, and ultimately preventing kidney fibrosis.

## Results

### Kidney MΦs upregulate AIF-1 expression following kidney injury.

**Figure 1:**
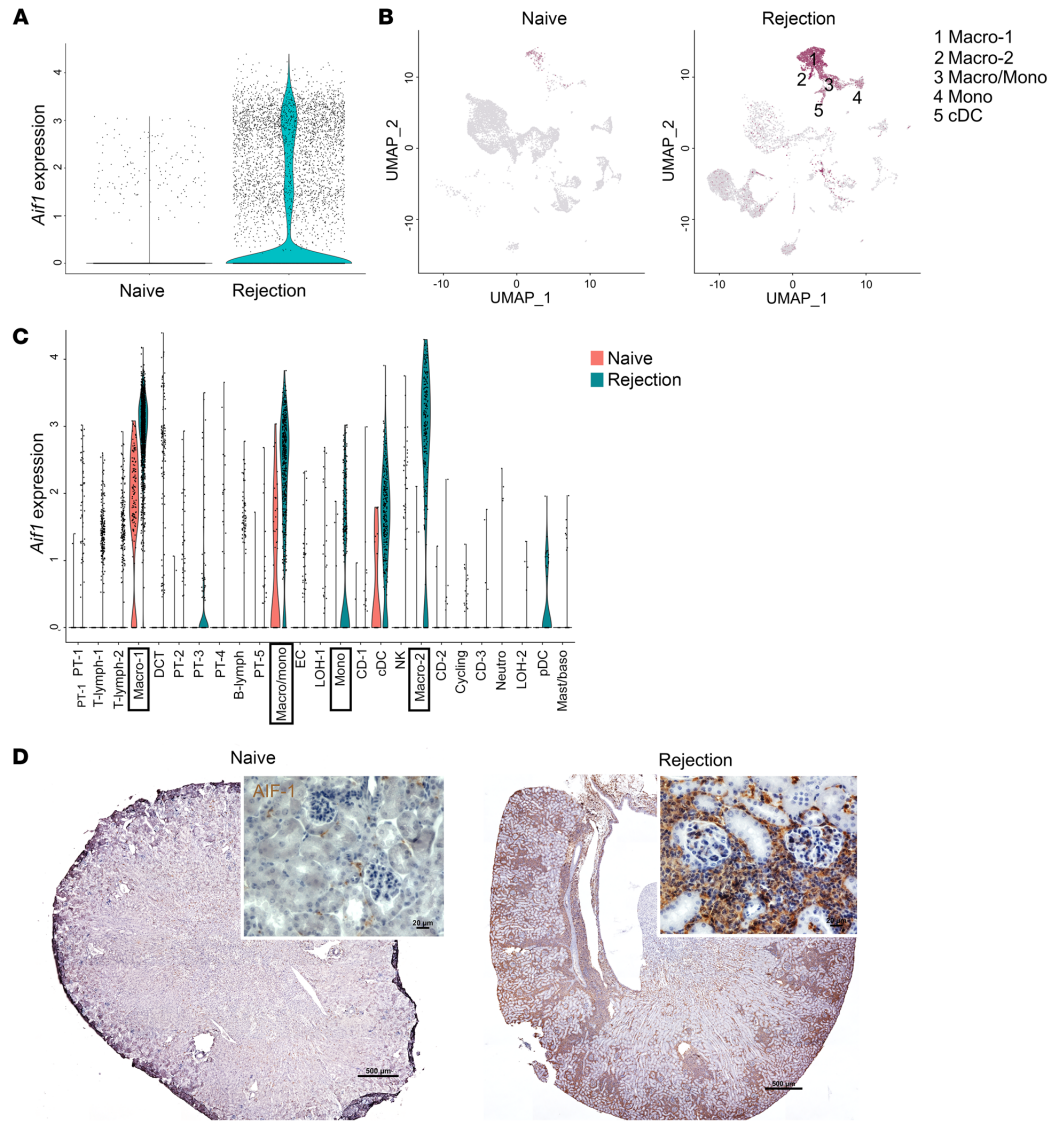
Kidney MΦs upregulate AIF-1 following kidney injury. (**A**–**C**) Single-cell sequencing analysis of *Aif1*-expressing cells in murine allogeneic kidney transplantation. On day 15 after transplantation (BALB/c kidneys transplanted into bilateral nephrectomized B6 recipients), and naive, unmanipulated BALB/c kidneys were used as controls (*n* = 2 each). (**A**) Violin plot showing total *Aif1* expression levels in naive and rejection kidneys. (**B**) Feature plots for naive and rejecting kidneys showing that myeloid cell subsets were the predominant cell populations expressing *Aif1*. (**C**) Violin plots showing *Aif1* expression levels by cell type in both naive and rejection samples. PT, proximal tubule; T-lymph, T lymphocyte; Macro, MΦ; DCT, distal convoluted tubule; B-lymph, B lymphocyte; Macro/Mono, MΦ/monocyte; EC, endothelial cell; LOH, loop of Henle; CD, collecting duct; cDC, conventional DC; Neutro, neutrophil; pDC, plasmacytoid DC; Mast/Baso, mast cell/basophil. (**D**) Immunohistochemical staining for AIF-1 (brown) in naive kidneys and rejecting kidneys at day 15 after transplantation. Images are representative of 3 each. Original magnification, ×100 for stitch images; ×400 (insets). Scale bars: 500 μm and 20 μm, respectively.

We have previously interrogated the immune cell landscape of MΦ-mediated inflammation in an allogeneic BALB/c-to-B6 murine kidney transplantation model using single-cell RNA-Seq (scRNA-Seq) (26). Using the same dataset, we assessed the expression pattern of *Aif1* in the kidney allograft during rejection in comparison with that in naive control kidneys. As shown in Figure 1A, *Aif1* expression was markedly increased in kidneys undergoing rejection. Feature plots showed *Aif1* transcripts localized largely to MΦ and monocyte subpopulations in rejecting kidneys, whereas naive kidneys had few MΦs and monocytes and lower per cell Aif1 transcript levels (Figure 1B). As shown in Figure 1C, MΦs from rejecting kidneys expressed the highest levels of *Aif1* transcripts (outlined by black rectangles). We next performed immunostaining for AIF-1 on control naive and rejecting kidneys (Figure 1D). We noted diffuse AIF-1 staining in the cortex of rejecting kidneys but not in controls. The presence of such a large number of MΦs after renal transplantation was a consequence of both I/RI and alloimmunity. In the clinical setting, chronic allograft dysfunction is characterized by diffuse MΦ infiltration and IF (27, 28). However, murine models are significantly limited in parsing out the individual effects of ischemia and alloimmunity in mediating chronic allograft nephropathy. Therefore, to investigate specifically the role of AIF-1 in kidney ischemia and ensuing fibrosis, we used a model of I/RI-mediated kidney fibrosis without alloimmunity in our subsequent experiments.

Eight- to 12-week-old mice underwent unilateral left kidney ischemia for 30 minutes followed by contralateral nonischemic right kidney nephrectomy approximately 48 hours before planned euthanasia. We intentionally delayed nephrectomy to optimize animal survival, while allowing the initial ischemic injury to result in maladaptive repair and fibrosis (29). The 48-hour interval between contralateral nephrectomy and sacrifice also ensured that the terminal serum creatinine reflected only the function of the ischemic kidney.

**Figure 2:**
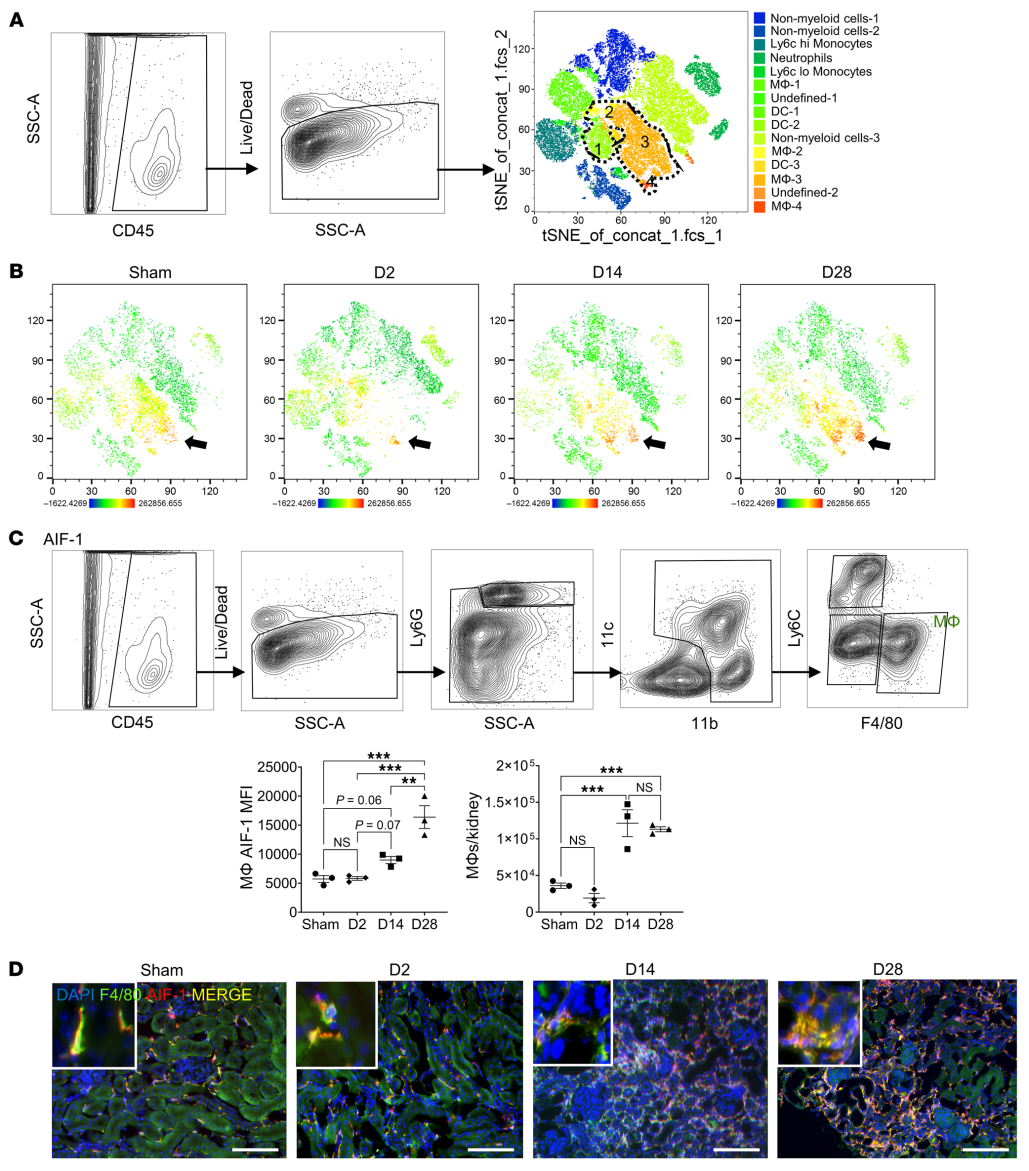
Kinetics of AIF-1 expression and MΦ infiltration in the kidney after I/RI. B6 mice underwent unilateral left kidney I/RI or sham surgery, and kidneys were collected at day 2, day 14 and day 28 after I/RI. Flow cytometry–based cell-type annotation and AIF-1 expression are shown in **A**–**C** ( *n* = 3 for each group). (**A**) Gating strategy and *t*-SNE plot gated on CD45^+^ live cells from all groups and all time points, showing 14 immune cell clusters. Cell clusters encircled with a dotted line were annotated as MΦs (MΦs 1–4). concat., concatenated; fcs, flow cytometry standard. (**B**) *t*-SNE plots separated by sham-operation, day 2 (D2), day 14 (D14), and day 28 (D28) showing AIF-1 expression. (**C**) Gating strategy for kidney MΦs. Graphs show kidney MΦ AIF-1 MFI and total MΦ numbers per kidney. Each symbol represents data from an individual mouse. Data indicate the mean ± SEM. **P < 0.01 and ***P < 0.001, by 1-way ANOVA with Tukey’s multiple-comparison test. (**D**) Immunofluorescence staining of kidney sections for F4/80 and AIF-1. Images are representative of 3 mice for sham, 6 mice for day 2 and day 14 after I/RI, and 8 mice for day 28 after IRI. Original magnification, ×200 (hpf, ×200); ×400 (inset). Scale bars: 100 μm. SSC-A, side scatter area.

Using this I/RI model, we first investigated the kinetics of (a) AIF-1 expression in MΦs and (b) MΦ infiltration into the ischemic kidney. We used flow cytometry to quantify MΦs and their AIF-1 MFI over the course of 28 days after I/RI, and, using immunofluorescence, we validated the cellular localization of AIF-1. Unsupervised clustering of CD45^+^ live cells from kidneys of mice after sham procedure or I/RI (day 2, day 14, and day 28) generated 14 different cell clusters (Figure 2A). Of these, 4 subpopulations were annotated as MΦs (MΦs 1–4, outlined by the dotted line), given their universal expression of the canonical MΦ markers F4/80 and CD11b (Supplemental Figure 1; supplemental material available online with this article; https://doi.org/10.1172/JCI185146DS1). A heatmap showing expression patterns of additional markers (CD11c, CD86, MHC II, Ly6G, CD206, and Ly6c) within these MΦ subpopulations is shown in Supplemental Figure 1. We then determined the localization of AIF-1 within immune cells using *t*-distributed stochastic neighbor embedding (*t*-SNE) plots. We found AIF-1 to be expressed within MΦ 1–4 subsets (Figure 2B, denoted by the black arrow) at all time points after I/RI as well as in kidneys from sham-operated mice. However, AIF-1 expression at day 28 after I/RI was more pronounced than in sham-operated animals or at other earlier time points after I/RI. Of note, the most robust expression was seen in subpopulations MΦ-3 and MΦ-4, both of which also expressed a high level of MHC II, whereas the MΦ-4 subpopulation expressed the highest levels of CD86 (Supplemental Figure 1). For subsequent quantification of AIF-1 levels by MFI and total MΦ counts, kidney MΦs were gated as CD45^+^Ly6G^–^CD11b^+^Ly6C^–^F4/80^+^ cells (Figure 2C). Total MΦ AIF-1 MFI at day 28 after I/RI was significantly higher in comparison with sham operation or other earlier post-I/RI time points. A trend toward increased expression was also observed at day 14 but not at day 2 after I/RI. Interestingly, MΦ infiltration appeared to persist long after the original ischemic insult in this model. Initially, there was a slight decline in total MΦ numbers at day 2 after-I/RI, likely due to ischemic injury and death of resident MΦs themselves. However, at day 14, there was a significant increase (~3-fold) in the number of MΦs in the kidney, likely from the circulation, and this elevation persisted to day 28 after I/RI. These findings were further confirmed with immunofluorescence staining, which showed coexpression of AIF-1 and F4/80 in the kidney in both sham-operated and I/RI mice (Figure 2D, merged image). As expected, all kidneys from mice 28 days after I/RI showed marked fibrosis and atrophy (as described below). However, AIF-1^+^F4/80^+^ MΦs appeared to be diffusely distributed throughout the kidney cortex and did not colocalize with specific areas of fibrosis or atrophy. In conclusion, robust and persistent AIF-1 expression was induced in ischemic injury–associated kidney MΦs.

### Aif1 deletion protects the kidney from IFTA after I/RI.

**Figure 3:**
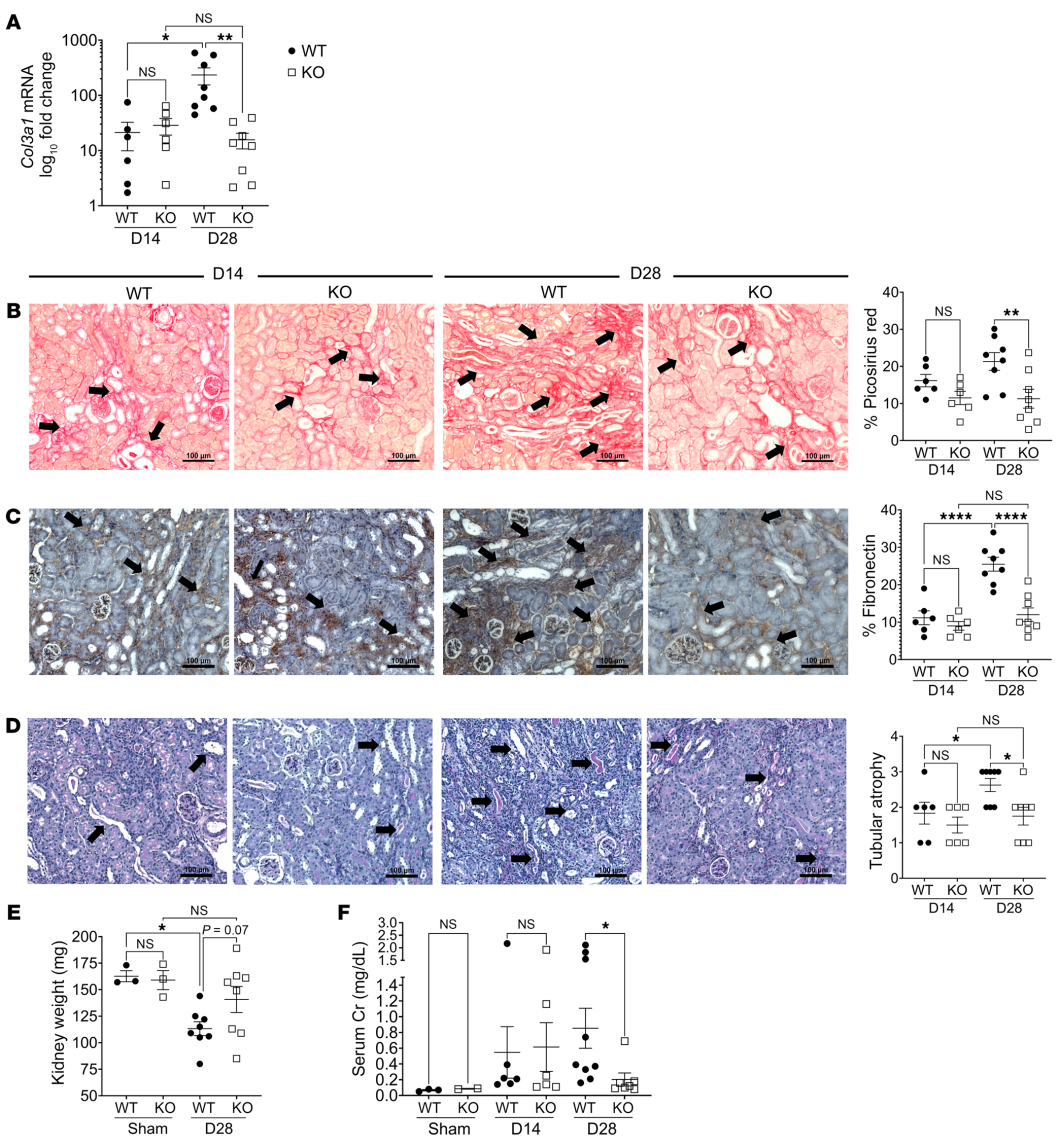
*Aif1* KO attenuates kidney fibrosis and atrophy. (**A**) *Col3a1* mRNA expression in the indicated groups was measured by RT-qPCR. Data are presented on a log_10_ axis. (**B**) Images and quantification graph of Picrosirius red staining of the kidney for the indicated groups. (**C**) Images and quantification graph of fibronectin staining of the kidney in indicated groups. (**D**) H&E-stained images of kidney sections and quantification graph of TA for the indicated groups. (**E**) Kidney weights (in mg) for the indicated groups. (**F**) Kidney function by serum creatinine (Cr) levels for the indicated groups. For **A**–**F**, each symbol represents data from an individual mouse. For **B**–**D**, images are representative of 6 each for WT and KO for day 14 after I/RI and 8 each for WT and KO for day 28 after I/RI. Original magnification, ×200. Scale bars: 100 μm. Black arrows denote positive areas. Data indicate the mean ± SEM. *P < 0.05, **P < 0.01, and ****P < 0.0001, by 1-way ANOVA with Tukey’s multiple-comparison test (**A**–**F**). KO, *Aif1*-KO.

Noting the strong association of AIF-1 with persistent MΦ infiltration following I/RI, we next examined the effects of *Aif1* deletion on the development of kidney fibrosis and atrophy following I/RI. Both *Aif1^+/+^* (WT) and *Aif1^–/–^* (KO) mice underwent I/RI followed by interval nephrectomy as described above. We first measured the expression of collagen type III α 1 chain gene (*Col3a1*), as it has been associated with multiple fibrotic diseases including kidney fibrosis (30). As shown in Figure 3A, at day 28 after I/RI, kidneys from KO mice expressed significantly lower *Col3a1* mRNA levels than did those from WT mice, whereas no difference was observed in *Col3a1* expression on day 14 after I/RI (Figure 3A) or at baseline (Supplemental Figure 2A). Furthermore, in WT mice, *Col3a1* expression increased between day 14 and day 28 after I/RI, suggesting continued progression of fibrosis with time in WT mice. However, in KO mice, there was no further increase in *Col3a1* expression between day 14 and day 28 after I/RI, suggesting that the fibrosis progression was halted in KO mice. We next examined the development of kidney IF by Picrosirius red staining and by staining for fibronectin, another marker of fibrosis in addition to *Col3a1*. As expected, we found that both Picrosirius red and fibronectin staining were minimal in nonischemic kidneys (Supplemental Figure 2B). Quantification of both Picrosirius red and fibronectin staining demonstrated significantly less fibrosis on day 28 after I/RI in kidneys from KO mice in comparison with those from WT mice (Figure 3, B and C). We also examined the degree of tubular atrophy (TA) by H&E staining. As shown in Figure 3D, KO mice had significantly less TA than did WT mice on day 28 after I/RI. Consistently across all parameters examined, kidney IFTA continued to worsen from day 14 to day 28 in WT mice, whereas in KO mice, this progression was halted beyond day 14 (Figure 3, A–D). Last, we measured kidney weight (Figure 3E) and serial serum creatinine levels (Figure 3F) as overall measurements for nephron mass and kidney function. As shown in Figure 3E, on day 28 after I/RI, KO kidneys weights were similar to those their sham counterparts, whereas WT kidneys weighted significantly less than did their sham counterparts. Consistently, serum creatinine levels are also significantly lower in KO mice that in WT mice in day 28 (Figure 3F). Collectively, these findings indicate that *Aif1* deletion protected kidneys from the development of IFTA and promoted superior kidney function at day 28 following I/RI.

It is important to note that the severity of early injury and renal inflammation, as measured by serum creatinine, tubular injury markers (Ngal and Kim1), histological injury score, inflammatory cytokines (Il1b and Nos2) expression and CD86+ inflammatory MΦ infiltration (Supplemental Figure 3), did not differ between WT and KO groups.. Therefore, the protective effect from *Aif1* deletion appeared to be a late, rather than an early, effect.

As an important control, we validated that there were no baseline differences between the WT and KO mice in terms of their kidney MΦ expression of antiinflammatory (arginase 1 [Arg-1, *Arg1*], chitinase-like 3 [Chil3, *Chil3*, also known as *Ym1*], *Il10*) and proinflammatory (*Il1b*, *Il6*, NLR family, pyrin domain–containing 3 [*Nlrp3*]) gene transcript expression (Supplemental Figure 4A), as well as CD206 and CD86 expression in kidney (Supplemental Figure 4B) and splenic (Supplemental Figure 4C) MΦs. Therefore, an injury stimulus such as I/RI was required to enhance MΦ AIF-1 expression and its deleterious effects, but in an uninjured state, we found no effects of *Aif1* deletion on MΦ phenotype.

### Aif1^–/–^ MΦs play a dominant role in kidney protection following I/RI.

**Figure 4:**
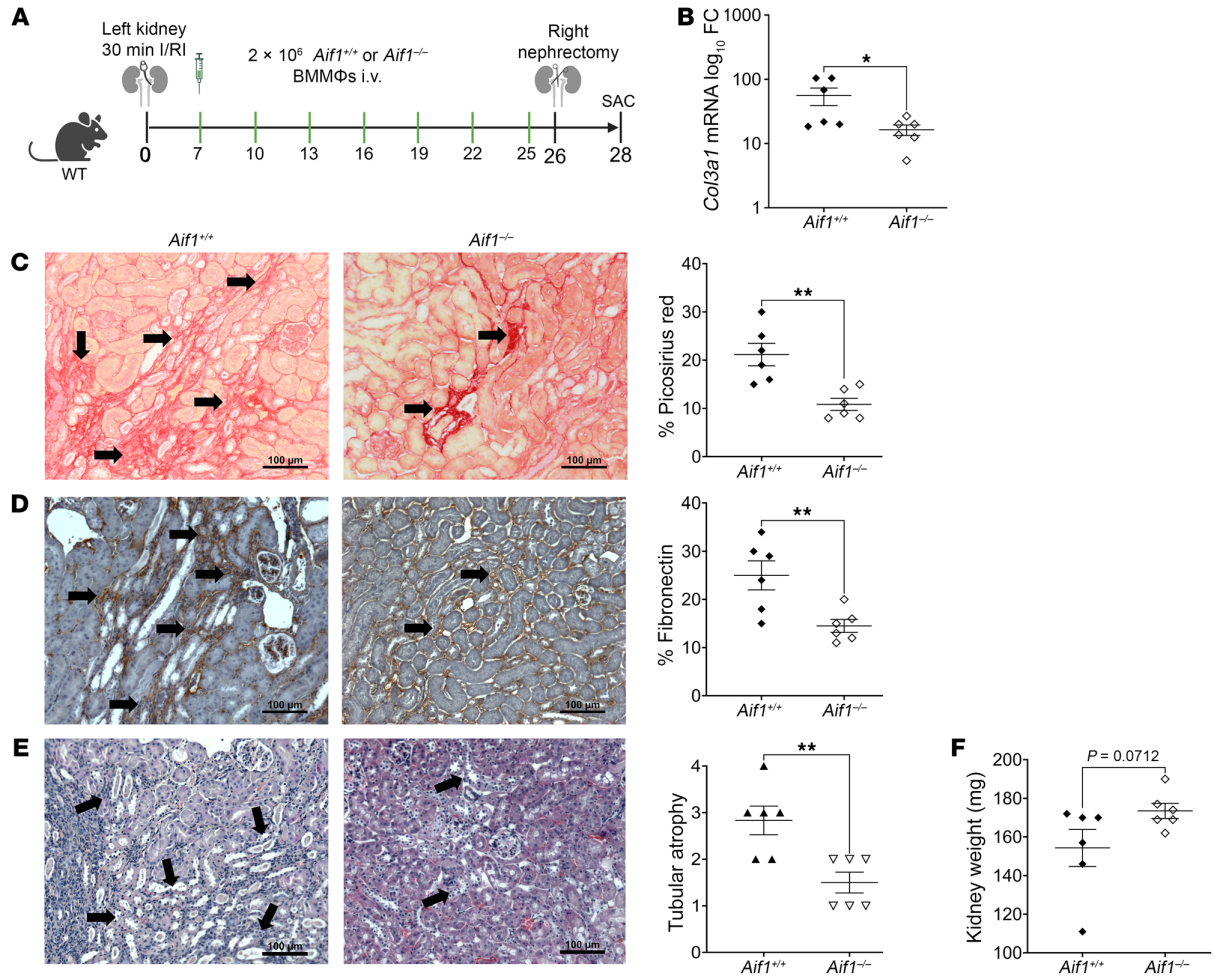
Adoptive transfer of *Aif1^–/–^* MΦs alleviates fibrosis following I/RI in WT mice. (**A**) Schema of the model for I/RI and adoptive transfer of *Aif1^+/+^* and *Aif1^–/–^* BMMΦs. SAC, sacrifice. (**B**) *Col3a1* mRNA expression in the indicated groups was measured by RT-qPCR. Data are presented on a log_10_ axis. (**C**) Images and quantification graph of Picrosirius red staining of the kidney for the indicated groups. (**D**) Images and quantification graph of fibronectin staining of the kidney in the indicated groups. (**E**) H&E-stained images of kidney sections and quantification graph of TA for the indicated groups. (**F**) Kidney weights are shown (in mg) for the indicated groups. For **B**–**F**, each symbol represents data from an individual mouse. For **C**–**E**, images are representative of 6 per group. Black arrows denote positive areas. Original magnification, ×200. Scale bars: 100 μm. Data indicate the mean ± SEM. *P < 0.05 and **P < 0.01, by unpaired, 2-tailed Student’s *t* test (**B**–**F**). SAC, sacrifice.

The predominant expression of AIF-1 in kidney MΦs (Figure 2) and the superior functional outcome in *Aif1^–/–^* mice following I/RI (Figure 3) led us to hypothesize that the lack of AIF-1 in kidney MΦs was the primary mechanism of protection against the development of IFTA after I/RI. To test our hypothesis, we induced I/RI in WT mice and, starting on day 7, adoptively transferred *Aif1^–/–^* bone marrow–derived MΦs (BMMΦs) at a dose of 2 × 10^6^ i.v. every 3 days until the mice were sacrificed on day 28 (Figure 4A). Adoptive transfer of *Aif1^+/+^* BMMΦs was similarly performed as a control. We started adoptive transfer on day 7 after I/RI to avoid altering early injury and inflammation, which were similar in both WT and KO groups (Supplemental Figure 3). Using congenic markers (CD45.1 for hosts and CD45.2 for adoptively transferred BMMΦs), we confirmed the presence of CD45.2^+^ MΦs in the kidney but noted that the number of host CD45.1^+^ MΦs predominated over that of the transferred CD45.2^+^ MΦs (Supplemental Figure 5, A–C). On day 28 after I/RI, kidneys from mice receiving *Aif1^–/–^* BMMΦs showed significantly lower levels of *Col3a1* mRNA expression (Figure 4B), Picrosirius red^+^ area (Figure 4C), fibronectin staining (Figure 4D), and TA (Figure 4E) in comparison with kidneys from mice that received *Aif1^+/+^* BMMΦs. Altogether, these findings revealed a trend toward higher kidney weights on day 28 in mice receiving *Aif1^–/–^* BMMΦs (Figure 4F). However, no notable difference in serum creatinine was observed (Supplemental Figure 6). Given the predominance of recipient MΦs (Supplemental Figure 5), it is conceivable that the lack of creatinine change at day 28 could reflect the few transferred MΦs relative to the host MΦs In addition to creatinine levels in mice being an inaccurate measurement of small differences in kidney function (31). Future experiments testing escalating doses of adoptively transferred MΦs may demonstrate further enhancement of kidney function on day 28 after I/RI. These data demonstrate that *Aif1^–/–^* BMMΦs played a protective role following kidney I/RI in a dominant fashion. Additionally, we also concluded that the significance of targeting AIF-1 predominantly stemmed from infiltrating MΦs from the circulation rather than kidney-resident MΦs, since we observed this protective effect with i.v. adoptive transfer of MΦs derived from BM precursors.

### Aif1^–/–^ MΦs play an obligatory role in kidney protection following I/RI.

**Figure 5:**
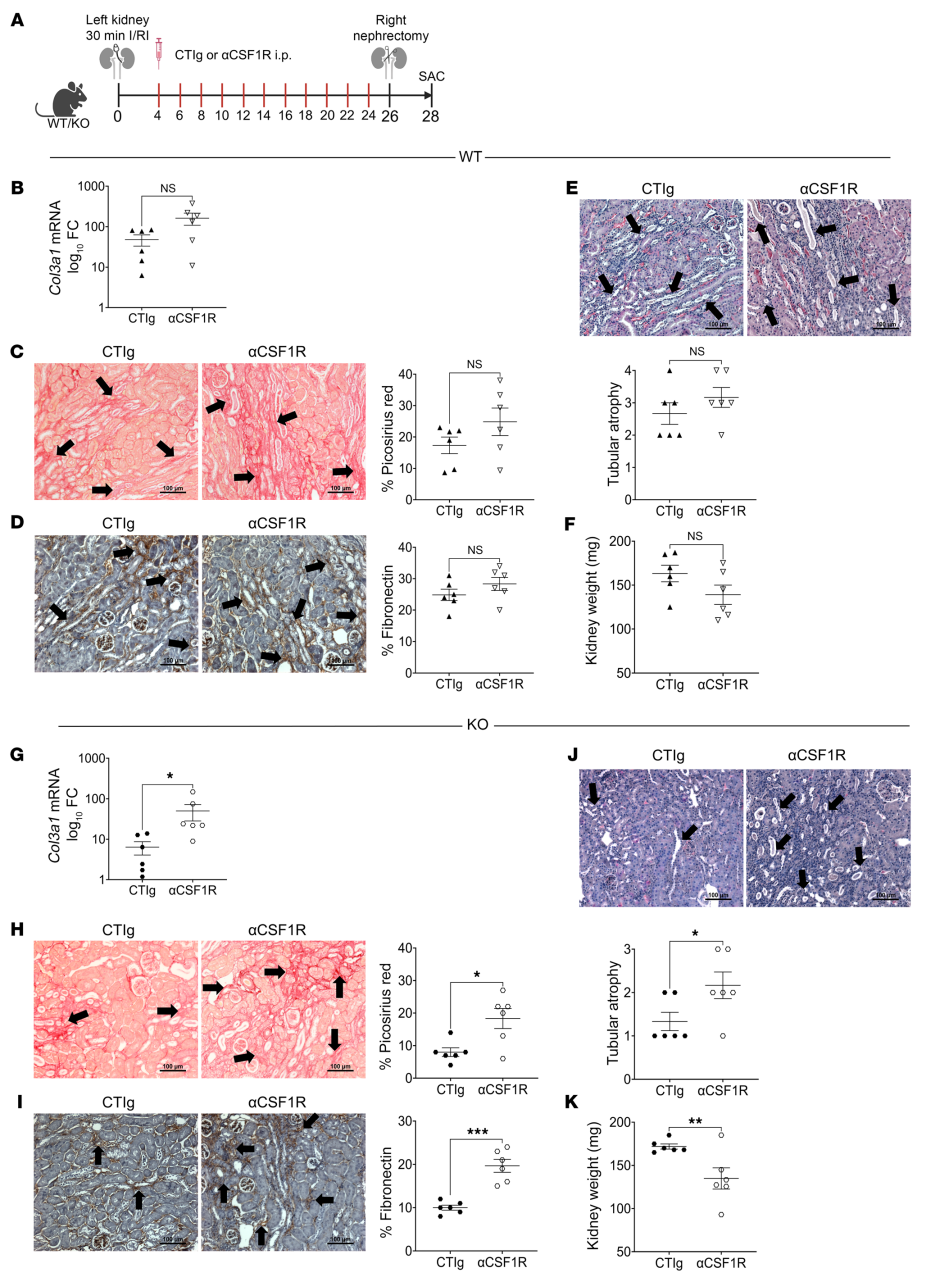
MΦ depletion in *Aif1*-KO mice worsens fibrosis and atrophy after I/RI. (**A**) Schema of the model for I/RI and administration of αCSF1R antibody or CTIg. (**B**–**F**) Data are from WT mice. (**B**) *Col3a1* mRNA expression in WT mice treated with CTIg and αCSF1R was measured by RT-qPCR. Data are presented on a log_10_ axis. (**C**) Images and quantification graph of Picrosirius red staining of the kidney for the indicated groups. (**D**) Images and quantification graph of fibronectin staining of the kidney for the indicated groups. (**E**) H&E-stained images of kidney sections and quantification graph of TA for the indicated groups. (**F**) Kidney weights (in mg) for the indicated groups. (**G**–**K**) Same as in **B**–**F**, except the data pertain to KO mice. In **B**–**K**, each symbol represents data from an individual mouse. For **C**–**E** and **H**–**J**, images are representative of 6 for each group. Data indicate the mean ± SEM. *P < 0.05, **P < 0.01, and ***P < 0.001, by unpaired, 2 tailed Student’s *t* test (**B**–**K**). Black arrows in the images denote positive areas. Scale bars: 100 μm.

To definitively attribute the superior kidney outcomes in KO mice after I/RI to kidney-infiltrating MΦs, we conducted a MΦ depletion experiment. Here, we depleted MΦs with a course of anti–colony-stimulating factor 1R (αCSF1R) in both WT and KO mice after I/RI, followed by examination on day 28 (Figure 5A). We chose day 4 after I/RI to begin αCSF1R treatment on the basis of the rationale that (a) depletion prior to I/RI would predominantly remove the kidney-resident MΦ subset (32), while in our model the protective effect of KO MΦs appeared to be exerted by circulating MΦs, and (b) during the early post-I/RI phase, MΦs likely exert a proinflammatory effect (10) in both KO and WT mice. Therefore, we wanted to minimize such a confounder by delaying our depletion.

We first determined the efficacy and specificity of αCSF1R treatment. As shown in Supplemental Figure 7A, in both WT and KO mice treated with αCSF1R (using the schedule in Figure 5A), at day 28 after I/RI, we observed a near-complete depletion of MΦs in the kidney, whereas other myeloid cells in the kidney (monocytes, neutrophils, and DCs) were minimally affected. MΦ depletion was further confirmed by immunofluorescence staining for F4/80 (Supplemental Figure 7B).

We next examined the kidney outcomes of MΦ depletion in WT and KO mice. In WT mice, no significant difference was observed in *Col3a1* mRNA expression (Figure 5B), Picrosirius red^+^ areas (Figure 5C), fibronectin staining (Figure 5D), TA (Figure 5E), kidney weights (Figure 5F), or serum creatinine levels (Supplemental Figure 8A) between kidneys from mice receiving αCSF1R or control Ig (CTIg). In contrast, in KO mice, kidneys from mice receiving αCSF1R showed significantly higher levels of *Col3a1* mRNA expression (Figure 5G), Picrosirius red^+^ areas (Figure 5H), fibronectin staining (Figure 5I), and TA (Figure 5J) on day 28 compared with kidneys from CTIg-treated mice, and, consequently, these kidneys had significantly reduced kidney weights (Figure 5K) and a trend toward higher serum creatinine levels (Supplemental Figure 8B) compared with their CTIg-treated counterparts.

Collectively, these data support the idea that *Aif1^–/–^* MΦs play an obligatory role in kidney protection following I/RI. Notably, depletion of MΦs in WT mice did not have a detrimental or protective effect following I/RI. This is likely because the αCSF1R-mediated depletion of heterogeneous and functionally dichotomous populations of MΦs in WT mice (namely, proinflammatory versus reparative; AIF-1–expressing versus –nonexpressing subsets) had a neutral end result.

### Aif1 deletion promotes a reparative phenotype in kidney MΦs in response to I/RI.

On the basis of the findings in the above MΦ adoptive transfer (Figure 4) and depletion (Figure 5) experiments, we hypothesized that the superior kidney function after I/RI was largely mediated by *Aif1*-KO MΦs. Before examining *Aif1*-KO MΦs in detail, we first sought to determine whether *Aif1* deficiency had any effect on non-MΦ immune cell populations after I/RI. As shown in Supplemental Figure 9, A and B, and Supplemental Figure 10, we observed no differences in non-MΦ immune cell populations between WT and KO mice kidneys after I/RI, including non-MΦ myeloid cells (monocytes, neutrophils, DCs) and T cells (CD4^+^ and CD8^+^). Additionally, we did not observe any difference in transcripts of T cell activation, differentiation, or effector function after I/RI (Supplemental Figure 9C).

Numerous studies have attempted to define mechanisms of MΦ proreparative functions (33–35). These functions can be broadly categorized as follows: (a) noninflammatory clearance of apoptotic cells (efferocytosis); (b) suppression of inflammation; and (c) tissue regeneration. Therefore, we evaluated the expression of molecules associated with these distinct proreparative functions in *Aif1^–/–^* kidney MΦs.

**Figure 6:**
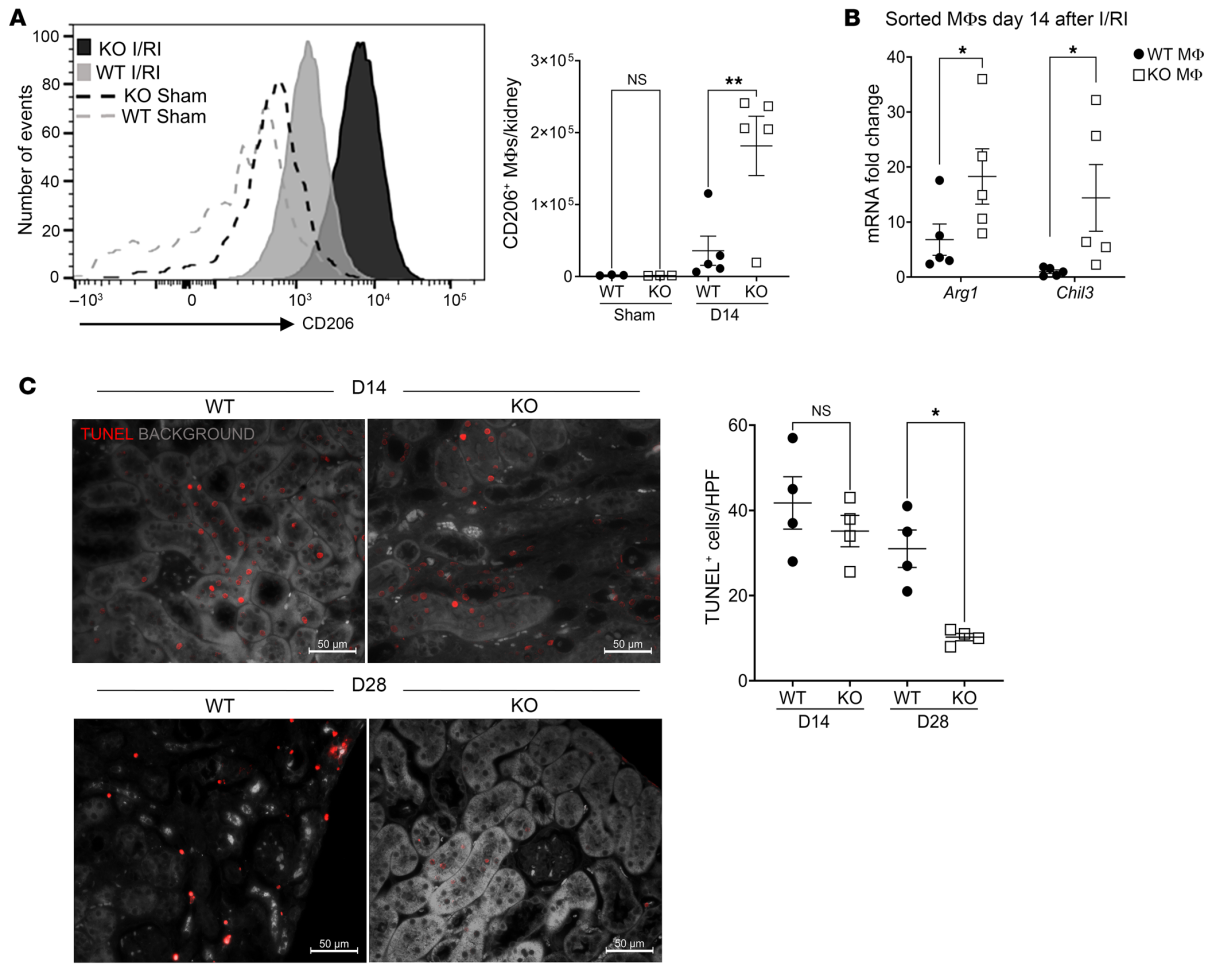
*Aif1* deletion enhances MΦ reparative functions. (**A**) Representative histogram and quantification graph showing CD206^+^ MΦs in the kidney at day 14 after I/RI or sham surgery in WT and KO mice. (**B**) Kidney MΦs were immunomagnetically purified on day 14 after I/RI or sham surgery, and gene expression for *Arg1* and *Chil3* was quantified by RT-qPCR. Expression was normalized to sorted MΦs from sham-operated mice. (**C**) Representative images of TUNEL staining of kidney sections and quantification graph for the indicated groups. Images are representative of 4 each for day 14 and day 28 after I/RI. Original magnification, ×200 (hpf, ×200). Scale bars: 50 μm. In **A**–**C**, each symbol represents data from an individual mouse. Data indicate the mean ± SEM. *P < 0.05 and **P < 0.01, by 1-way ANOVA with Tukey’s multiple-comparison test (**A**–**C**). KO, *Aif1*-KO.

First, we examined CD206, a mannose receptor involved in endocytosis and phagocytosis shown to play an important role in immune homeostasis (36). As shown in Figure 6A, the per-cell expression level (MFI) as well as total number of CD206-expressing kidney MΦs were significantly higher in KO mice than in WT mice on day 14 after I/RI. Second, we quantified the relative expression levels of *Arg1* and *Chil3*/*Ym1* transcripts in kidney MΦs (sorted for F4/80^+^ cells) on day 14 after I/RI. Arg-1 is a marker of alternatively activated MΦs shown to promote kidney tubular cell proliferation and repair (37). Chil3/Ym1 has also been associated with alternative MΦ activation, although its precise function is currently unclear (38). As shown in Figure 6B, in kidney MΦs isolated from KO mice, relative expression levels of *Arg1* and *Chil3* were significantly higher than in MΦs from WT mice on day 14 after I/RI (all expression levels were normalized to those of sham controls). Interestingly, in WT MΦs in which Arg-1 expression was already quite low, we did not find a further correlation of its expression with AIF-1 expression levels (Supplemental Figure 11).

Next, to assess the effect of these factors, we quantified the burden of tubular cell death in vivo by TUNEL staining following I/RI. We used background autofluorescence to highlight the normal kidney architecture. These TUNEL^+^ nuclei were primarily located in cells surrounding the tubular, thus likely representing dying RTECs. As shown in Figure 6C, at day 14 after I/RI, the burden of RTEC death (number of TUNEL^+^ cells per ×200 high-power field [hpf]) was comparable between KO and WT kidneys. However, by day 28, KO kidneys had significantly fewer TUNEL^+^ cells than did WT kidneys. We also examined cell death at an even earlier time point (day 2) after I/RI and noted a large number of TUNEL^+^ cells in both WT and KO mice, and no difference was observed between the groups (Supplemental Figure 12). From these data, we concluded that the persistence of TUNEL^+^ cells in kidneys from WT, but not KO, mice on day 28 was the result of failed repair in WT mice. To further explore downstream cellular targets of these reparative MΦs, we examined fibroblast activation by measuring α smooth muscle actin (α*sma*, also known as *acta2*) transcripts on day 28 after I/RI. We measured this in mice receiving *Aif1^+/+^* or *Aif1^–/–^* BMMΦs as in Figure 4. No differences were observed between the 2 groups (Supplemental Figure 13), which indicated that in our model, *Aif1* deletion did not alter MΦ-mediated myofibroblast activation.

Having observed that the absence of *Aif1* augmented polarization of MΦs to a reparative phenotype, we next decided to define the potential regulatory pathways altered by *Aif1* in kidney MΦs in vivo. As previously reported in the literature, the presumed time frame of the MΦ phenotypic switch after I/RI is between 3 and 5 days after ischemia (39). Therefore, we examined the gene transcription profile of sorted kidney MΦs on day 4 after I/RI using NanoString nCounter analysis. We performed pathway analysis on differentially expressed genes (DEGs) obtained upon comparison of KO MΦs with their WT counterparts after I/RI. We found that the Wnt/β-catenin signaling pathways were differentially regulated in KO versus WT MΦs (Supplemental Figure 14A). Excessive activation of Wnt signaling has been associated with CKD (40–42), and injured renal tubular cells have been shown to produce Wnt ligands (43, 44). We found that genes that activate the canonical Wnt/β-catenin signaling pathway (frizzled class receptor 4 [*Fzd4*], ref. 45; *Ctnnb1*, ref. 40; *Cdh1*, refs. 46, 47; *Jun*, refs. 48, 49) were downregulated in KO MΦs, whereas genes that inhibit Wnt/β-catenin signaling (*Fzd6*, ref. 50; secreted frizzled protein 4 [*Sfrp4*], ref. 51) were upregulated in KO MΦs. Additionally, examination of DEGs revealed that KO MΦs expressed higher levels of reparative markers (*Mrc2* , ref. 52; *Retnlb*/*Fizz1*, ref. 53; highlighted in orange in Supplemental Figure 14B) in comparison with WT MΦs. Conversely, KO MΦs downregulated genes associated with chronic inflammation and poor healing (*Ly6c1*, ref. 54; *Cxcl12*, ref. 55; *Gata2*, ref. 56; *Arf6*, ref. 57; *Peli1*, ref. 58; *Irf1*, ref. 59; *Calr*, ref. 60; highlighted in purple in Supplemental Figure 14B) compared with WT MΦs.

Collectively, our unbiased pathway analyses and exploration of the MΦ phenotype suggests that AIF-1/Wnt/β-catenin signaling probably directed the fate of MΦs toward reparative polarization, which was characterized by efficient efferocytosis and production of antiinflammatory humoral factors that together reduce the burden of RTEC death.

### Aif1^–/–^ MΦs protect RTECs during hypoxic injury in vitro.

**Figure 7:**
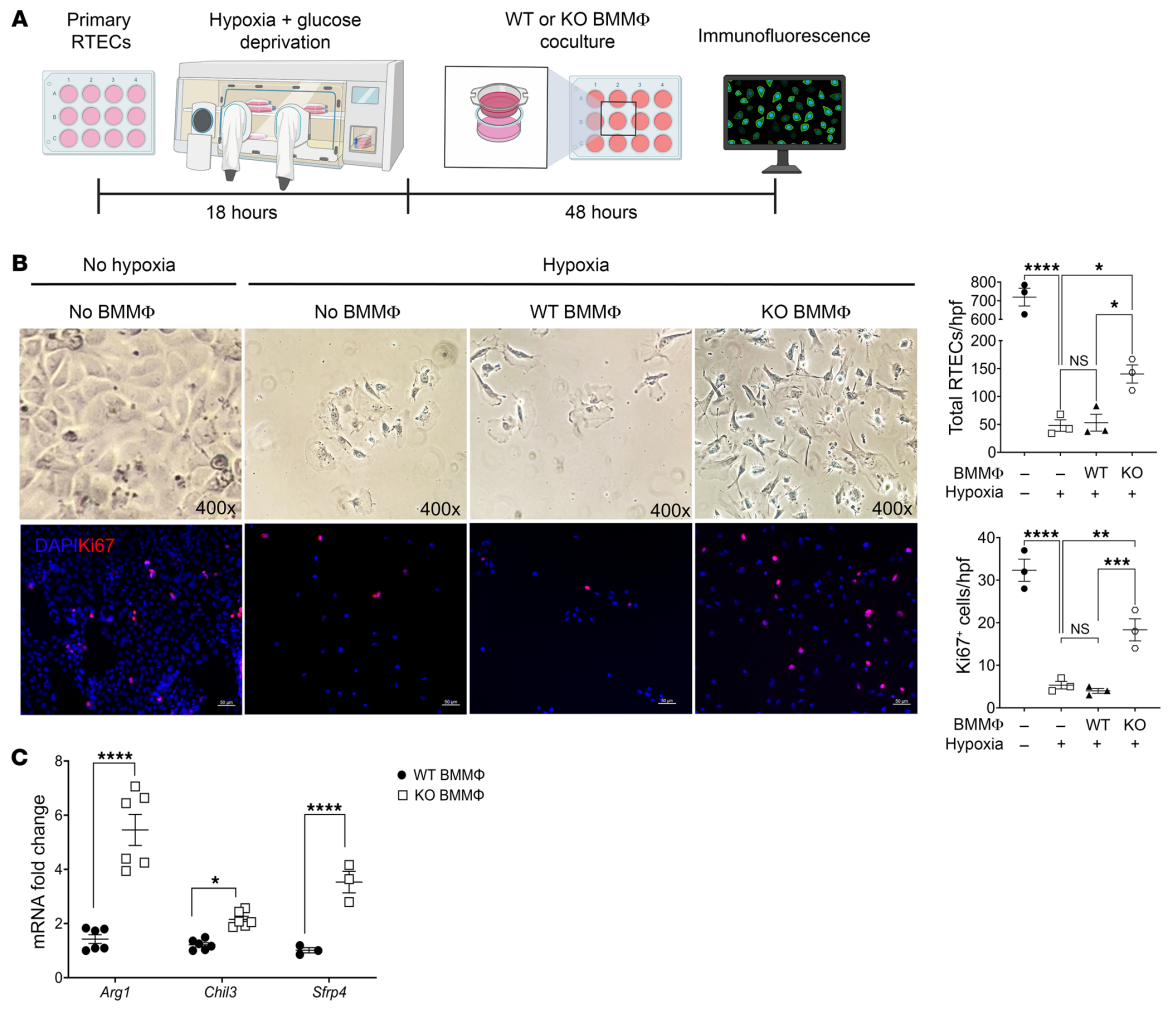
*Aif1^–/–^* MΦs protect RTECs from hypoxic injury in vitro. (**A**) Model of hypoxia and reoxygenation of RTECs followed by Transwell coculturing with BMMΦs from *Aif1*-KO and WT mice. (**B**) Upper panels: Representative Bright-field microscopy images of RTECs from the indicated groups. Original magnification, ×400. Lower panels: Representative immunofluorescence images with DAPI (blue) and Ki67 (red) staining of RTECs from the same groups. Original magnification, ×200. Scale bars: 50 μm. Images are representative of 3 for each group. Quantification graphs show total RTECs/hpf (DAPI^+^ cells) and Ki67^+^ cells/hpf. (**C**) WT and KO BMMΦs were treated with CM from RTECs subjected to 18-hour hypoxia followed by 48-hour reoxygenation. BMMΦs were cultured in the CM for a total of 48 hours and harvested for RT-qPCR analysis. Expression levels of *Arg1*, *Chil3*, and *Sfrp4* are shown. Data were normalized to expression levels in WT BMMΦs. For **B** and **C**, each symbol represents data from an individual mouse. Data indicate the mean ± SEM. *P < 0.05, **P < 0.01, ***P < 0.001, and ****P < 0.0001, by 1-way ANOVA with Tukey’s multiple-comparison test (**B** and **C**). Experiments were repeated twice.

To directly examine cellular interactions between MΦs and RTECs, we developed an in vitro BMMΦ-RTEC coculturing system. As shown in Figure 7A, primary RTECs underwent 18 hours of hypoxia plus simultaneous glucose deprivation (see Methods). At the time of reoxygenation, we added Transwells carrying either *Aif1^+/+^* or *Aif1^–/–^* BMMΦs and glucose-replete media to the culture for an additional 48 hours.

At the end of 48 hours of coculturing, we examined the RTECs for total cell numbers (by DAPI staining) and proliferation (by Ki67 staining). As shown in Figure 7B, following hypoxic injury and reoxygenation, RTECs cultured with *Aif1^–/–^* BMMΦs exhibited significantly higher total cell numbers as well as a higher number of Ki67-expressing cells in comparison with RTECs cultured alone or with *Aif1^+/+^* BMMΦs. As a control, we also examined the effect of *Aif1^+/+^* or *Aif1^–/–^* BMMΦs on RTECs that did not undergo hypoxia in our coculturing system and did not observe any differences in RTEC numbers or in their proliferation (Supplemental Figure 15).

We next examined the BMMΦ response in the presence of hypoxia-injured RTECs. To enhance cell numbers and replicates for the assessment, we cultured *Aif1^+/+^* or *Aif1^–/–^* BMMΦs directly in the conditioned media (CM) from RTECs undergoing hypoxia and reoxygenation (RTEC-hypoxic-CM) as described above. Interestingly, as shown in Figure 7C, *Aif1^–/–^* BMMΦs had significantly higher levels of *Arg1* and *Chil3* expression than *Aif1^+/+^* BMMΦs after exposure to RTEC-hypoxic-CM. We also examined the expression levels of *Sfrp4*, which is an inhibitor of Wnt/β-catenin signaling (61), a pathway that promotes MΦ polarization to a profibrotic phenotype (62). *Aif1^–/–^* BMMΦs expressed significantly higher levels of *Sfrp4* transcripts than did *Aif1^+/+^* BMMΦs after treatment with RTEC-hypoxic-CM. No baseline differences were observed when BMMΦs were cultured in media from RTECs that did not undergo hypoxia (data not shown). These in vitro findings support our in vivo findings in Figure 6B and Supplemental Figure 14.

Taken together, we conclude that *Aif1^–/–^* but not *Aif1^+/+^* BMMΦs, via inhibition of Wnt signaling, acquired a proreparative phenotype when exposed to secreted factors from hypoxia-injured RTECs and, consequently, promoted RTEC regeneration after injury.

### AIF1 is identified in MΦs in injured human kidneys.

**Figure 8:**
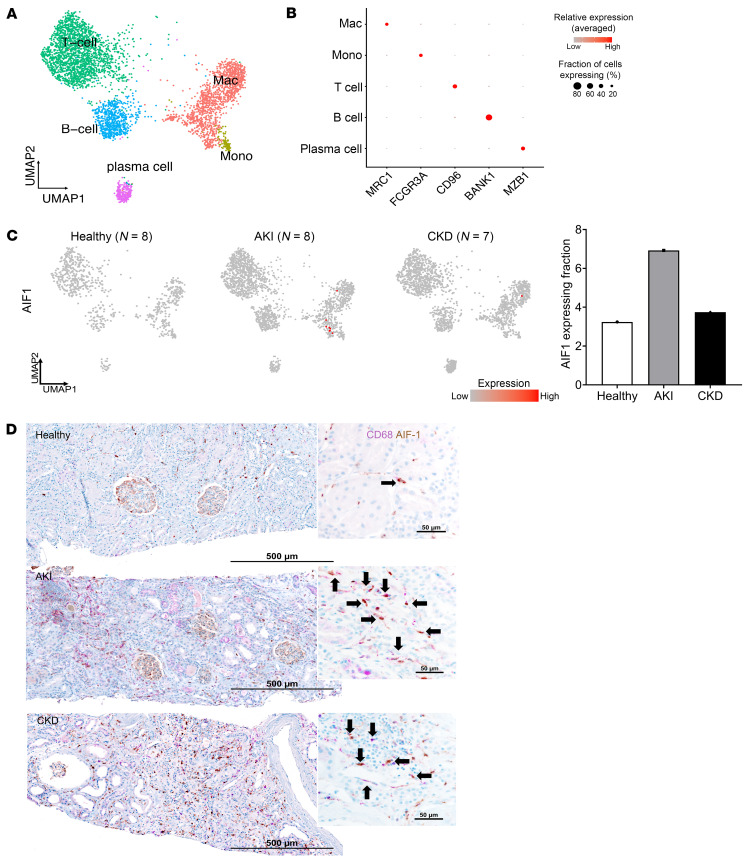
*Aif1* is expressed in human kidneys with AKI or CKD. (**A**–**C**) snRNA-Seq of kidney biopsy samples from healthy individuals (*n* = 8), patients with AKI (*n* = 8), and patients with CKD (*n* = 7). (**A**) A composite UMAP plot of all samples (total *n* = 23) showing 5 unique immune cell clusters. (**B**) Dot plot showing canonical marker expression used for cell-type annotation. (**C**) Individual feature plots separated by disease states (as indicated) showing *AIF1* expression, which was more pronounced in samples from patients with AKI or CKD than in healthy control samples within the monocyte/MΦ (Mono/Mac) and the CD163^+^ MΦ (CD163^+^ Mac) subsets. Quantification graph shows the percentage of *Aif1*-expressing cells among total (Mono/Mac and CD163^+^ Mac) cells by disease state. (**D**) Immunohistochemical staining for CD68 (purple) and AIF-1 (brown) in human biopsy samples of native kidneys (*n* = 3 each for healthy kidneys, AKI, and CKD). Original magnification, ×200 for stitch images; ×400 magnification. Scale bars: 500 μm and 50 μm, respectively. T cell, T lymphocyte; B cell, B lymphocyte. For **A**–**C**, results are based on data collected by the KPMP (63).

Having identified in murine models AIF-1 expression patterns in ischemia-provoked acute kidney injury (AKI), fibrosis, and atrophy as evidence of CKD development, and in kidney transplant rejection, we next examined human kidneys to measure AIF-1 expression in human kidney immune cells in response to injury. To this end, we generated a human kidney immune cell map by reanalyzing and integrating samples from 3 publicly available datasets (63–65) of single-nucleus RNA-Seq (snRNA-Seq) data (total of 23 samples; *n* = 8 healthy controls, *n* = 8 AKI samples, and *n* = 7 CKD samples) (Supplemental Table 1). We selected the immune cells on the basis of their expression of protein tyrosine phosphatase receptor type C (*PTPRC*), which encodes CD45. As shown in Figure 8, A and B, we observed all the major immune cell types in this integrated map. MΦ and monocyte subsets expressed their canonical markers *CD163*, *CD68*, and Fc γ receptor IIIa (*FCGR3A*). We subsequently generated feature plots for healthy, AKI, and CKD groups to visualize *AIF1* transcripts and quantified the percentage of *AIF1-*expressing cells among all CD163^+^ MΦs and monocytes/MΦs in each group (Figure 8C). We found that *AIF1* was primarily expressed in the MΦ and monocyte subsets, and that its expression was increased in both AKI and CKD kidney cells relative to healthy control kidney cells. We further confirmed this finding with immunohistochemical staining for AIF-1 and CD68 in 9 human kidney biopsies (*n* = 3 each for healthy controls, AKI, and CKD). As shown in Figure 8D, we found that AIF-1 was almost always coexpressed with CD68 in the tubulointerstitial compartment, therefore confirming its predominant expression by kidney MΦs. We further noted heightened AIF-1 expression levels in disease states (both AKI and CKD) compared with healthy controls. In addition, we established the relevance of AIF-1 in kidney transplant dysfunction, with AIF-1 and CD68 immunohistochemical staining of transplant kidney biopsies (*n* = 2 for healthy no-rejection allografts, *n* = 2 for chronic rejection, and *n* = 3 for acute rejection) (Supplemental Figure 16). In healthy no-rejection allografts, we observed scattered CD68^+^ cells without notable AIF-1 staining through the interstitium and some CD68 and AIF-1 costaining within the glomeruli. In contrast, in acute or chronic rejection biopsies, we observed dense infiltration of MΦs (CD68^+^ cells) with striking AIF-1 staining throughout the cortex, involving both the tubulointerstitial and glomerular compartments.

Collectively, these data confirm the association of AIF-1 with injury in both native and transplanted kidneys in humans and indicate the need for future studies targeting AIF-1 to ameliorate MΦ-mediated kidney injury in humans.

## Discussion

In this study, we first demonstrated that 2 distinct etiologies of kidney injury, kidney transplant rejection and ischemia reperfusion, were both characterized by AIF-1 upregulation in kidney MΦs. For subsequent experiments, we chose to investigate the role of AIF-1 in ischemia injury in the native kidney. We showed that the absence of *Aif1* altered Wnt/β-catenin signaling pathways and induced proreparative functions in kidney MΦs. Therefore, *Aif1* deletion protected hosts from progression of kidney injury from I/RI to IFTA. Adoptive transfer of *Aif1^–/–^* BMMΦs into WT mice provided dominant protection against I/RI, whereas depletion of MΦs in KO mice reversed this protection. These results collectively support the conclusion that AIF-1 plays a critical role in directing the phenotype and function of kidney MΦs following I/RI.

Although our findings were predominantly derived from native kidneys, we believe that they are directly relevant in the setting of kidney transplantation. First, AIF-1 expression was enhanced by ongoing alloimmunity, similar to that following I/RI, in both rodents (Figure 1) and humans (Supplemental Figure 16). Mechanisms for this upregulation and its maintenance are currently unclear, but is likely attributable to a high IFN-γ state such as from activated adaptive (NK, T cells) (66, 67) and innate (68, 69) immune cells. The expression kinetics of AIF-1 and its signaling in kidney allograft MΦs in the longitudinal post-transplantation course are currently being actively investigated in our laboratory. Second, ischemia is inevitable in kidney transplantation, including ischemia induced by calcineurin inhibitors necessary for chronic immunosuppression. Therefore, much like in the native kidneys, AIF-1 expression is also likely upregulated under these circumstances. As chronic allograft nephropathy (CAN), characterized by kidney fibrosis, is a leading cause of long-term kidney allograft failure, we believe that understanding the role of MΦ AIF-1 in promoting kidney fibrosis may reveal therapeutic targets effective for halting fibrosis and prolonging kidney transplant survival.

Despite the use of a global *Aif1*-KO model, we were able to attribute the protective effect of *Aif1* deletion specifically to kidney-infiltrating MΦs from circulation (a) by demonstrating a superior outcome with adoptive transfer of *Aif1^–/–^* BMMΦs into WT mice following I/RI and (b) the reciprocal experiment showing worsening of kidney IFTA with depletion of these protective MΦs in KO mice. Having observed the protective effect with adoptive transfer of *Aif1^–/–^* BMMΦs derived from BM precursors (Figure 4), we speculate that the inflammatory MΦs with high expression of AIF-1 as described in Figure 2 were of BM origin rather than a kidney-resident population. Such an infiltrating population has been previously implicated in chronic inflammation and failed repair (70), and AIF-1 is a potential marker for such inflammation. Definitive determination of the MΦ origin is limited with our current model. Our future directions include further determining the cell origin in an allogeneic kidney transplant model with congenically labeled cells and testing the protective effect of MΦ *Aif1* deficiency with variable durations of warm and cold ischemia.

Using TUNEL staining, we observed similar early tubular cell death in both KO and WT kidneys after I/RI (Supplemental Figure 12), but by day 28, a significantly higher degree of tubular cell death was evident in WT kidneys (Figure 6C). Such continued cell death has been previously described in cases of prolonged ischemia and is likely a feature of failed repair in AKI-to-CKD transition (71, 72). This, coupled with our in vitro findings that KO MΦs facilitated a reduction in hypoxia-induced RTEC death and increased their proliferation, suggests that ongoing RTEC proliferation in excess of cell death prevented the development of TA in kidneys from KO mice. Additionally, the protective effect of *Aif1^–/–^* BMMΦs on RTECs was exerted in the absence of cell-to-cell contact (Figure 7). Therefore, we conclude that proreparative functions of KO MΦs were mediated by secretory factors and that localization of MΦs to areas of fibrosis or injured tubular cells was probably not necessary for this function. Unlike WT MΦs, the Arg-1 produced by KO MΦs (Figure 6B and Figure 7C) promoted metabolism of arginine to produce urea and ornithine, which were then converted by ornithine decarboxylase enzyme to polyamines (73). As previously published, these polyamines exert a wide array of antiinflammatory effects such as reduction of IL-1β and TNF-α (74), as well as repair effects such as angiogenesis (75) and renal tubular cell proliferation (76).

On examining the transcriptional landscape of I/RI MΦs in the kidney (Supplemental Figure 14), we found that genes associated with Wnt/β-catenin signaling activation were upregulated in MΦs from kidneys in WT but not KO mice. A diverse array of kidney injury models have shown that upregulation of the renal Wnt/β-catenin pathway and its sustained activation leads to detrimental kidney outcomes (43, 44, 77). Specifically in I/RI, the Wnt family of proteins are produced by injured renal tubular cells, and through their binding to cell-surface receptors of the Fzd family, they promote signaling via β-catenin (43). Wnt signaling in MΦs has been shown to control their polarization (78). We found that KO MΦs upregulated genes that inhibit Wnt signaling (e.g., *Fzd6*, *Sfrp4*), whereas WT MΦs upregulated genes associated with canonical Wnt signaling activation (e.g. *Cdh1*, *Ctnnb1*). Therefore, we conclude that AIF-1 promoted canonical Wnt signaling to promote the polarization of MΦs to a profibrotic phenotype. Therefore, AIF-1 has the potential to refine the alternatively activated MΦ phenotype such that it uncouples the reparative from the profibrotic function of these cells.

*Aif1* silencing RNA (siRNA) has been previously tested in a murine model of autoimmune diabetes, in which a single i.p. injection of *Aif1* siRNA effectively restrained the expression of AIF-1 in pancreatic MΦs for 7 days (22). Therefore, *Aif1* siRNA could conceivably be used to mitigate kidney I/RI by promoting the reparative potential of MΦs and preventing AKI-to-CKD transition. In kidney transplantation, we foresee that targeting AIF-1 can mitigate the adverse effects of kidney ischemia during organ preservation and ultimately prolong kidney allograft survival.

In summary, we have demonstrated that altering the AIF-1/Wnt/β-catenin pathway enhanced kidney MΦ proregenerative functions and ultimately reduced kidney fibrosis and preserved kidney function following I/RI. Therefore, AIF-1 is a promising therapeutic target for the prevention of progression to kidney fibrosis and atrophy, in which a hemodynamic insult to the kidney is inevitable.

There are potential limitations to our current study. First, it is important to note that αCSF1R depleted all MΦs. As shown in Figure 2A and Supplemental Figure 1, after injury, MΦs were a heterogenous group with varying functions, therefore a more selective depletion strategy could potentially be more definitive. Second, while AIF-1 expression in MΦs was the primary focus of this study, our unsupervised approach as shown in the *t*-SNE plot (Figure 2A) and heatmap (Supplemental Figure 1) also found a small AIF-1–expressing DC population, annotated as DC-3 due to its expression of CD11c. Additionally, AIF-1 expression has been described in nonimmune cells such as podocytes (79), vascular smooth muscle cells (80), and endothelial cells (81). While our depletion and adoptive transfer experiments strongly support the notion that MΦs are the predominant cell population in which AIF-1 manifests its function, future studies utilizing cell-specific *Aif1* deletion will be necessary to elucidate the functional significance of AIF-1 in other cell types.

## Methods

### Sex as a biological variable.

Our study exclusively examined male mice, as female sex has been shown to be protective against I/RI (82). Further studies would be required to model injury severity using a longer warm ischemia duration to cause a degree of injury similar to that induced in the male mice in this study with 30 minutes of ischemia. However, we expect the findings to be relevant to the female sex as well.

### Mice.

*Aif1^–/–^* C57BL/6J (B6) mice were obtained from Edward B Thorp (Northwestern University, Chicago, Illinois, USA) (83). The mice were subsequently bred and housed at Duke University. Male 6- to 10-week-old B6 mice were purchased from The Jackson Laboratory. Mice were age-matched for all experiments. Each experiment was performed on a cage of animals, which served as single experimental unit. Two to 3 cages of mice were used per experiment to achieve the desired number of animals per group. For mice that underwent I/RI, the sample size was planned assuming 20% attrition due to post-procedure-related complications. For no- to low-variability groups such as the sham-operated mice group, 3–4 mice per group were considered adequate. For higher-variability groups, a larger number of mice (*n* = 6–8) were used. Sample sizes were not calculated a priori on the basis of a specific primary outcome. All animals that died prior to the intended date of euthanasia were excluded. No randomization was performed to allocate mice to the control or treatment groups. Confounders were minimized by (a) age matching all mice in each experiment, (b) performing I/RI in alternating sequence between the control and treatment groups, (c) using the same pedicle clamps for I/RI across the comparison groups, and (d) housing all animals in the same facility.

### Warm kidney I/RI.

Mice (8–12 weeks of age) were given buprenorphine SR for analgesia followed ketamine/xylazine for anesthesia and were then shaved and cleaned using betadine. Once the left renal pedicle was exposed, ischemia was induced by placing a pedicle clamp for 30 minutes. Throughout the procedure, the mice were maintained at 36°C–37°C on a temperature-controlled heating pad. After surgery, 250 μL saline was administered s.c. For mice that were sacrificed on day 14 and day 28, contralateral (right) nephrectomy was performed 48 hours prior to assessments. For the day-2 time point, right nephrectomy was performed at the same time as the left renal pedicle clamp placement (day 0).

### Depletion of kidney MΦs.

Mice were treated with αCSF1R antibody (BioXCell, clone AFS98) or CTIg (BioXCell, Rat IgG2a) via i.p. injection. Treatment was started on day 4 after I/RI. The first dose was 50 mg/kg followed by 25 mg/kg every other day until the day of sacrifice.

### BMMΦ generation.

BM cells were obtained from the femur and tibia of 6- to 10-week-old *Aif1^+/+^* and *Aif1^–/–-^* mice. These cells were grown in culture media with DMEM (catalog 11320033, Gibco, Thermo Fisher Scientific), 10% FBS (catalog A5256701, Gibco, Thermo Fisher Scientific), 1% penicillin-streptomycin (catalog 15140122, Gibco, Thermo Fisher Scientific), and 20 ng/mL M-CSF (catalog 416-ML, R&D Systems) for 7–10 days before use in experiments.

### Adoptive transfer of Aif1^–/–^ BMMΦs.

BM cells from *Aif1^–/–^* mice were cultured in macrophage-CSF–containing (M-CSF–containing) media as described above. On day 7 or day 10, the BM cells were harvested using a cell scraper and resuspended in saline at a concentration of 2 × 10^4^ cells/μL. Mice were i.v. injected with 2 × 10^6^ BMMΦs every 3 days. Treatment was started on day 7 after I/RI and continued until the day of sacrifice.

### Adoptive transfer of CD45.2 BMMΦs into CD45.1 recipients.

B6.SJL-Ptprca Pepcb/BoyJ (CD45.1) mice underwent left kidney I/RI for 30 minutes followed by adoptive transfer of 2 × 10^6^ *Aif1^–/–^* BMMΦs (CD45.2) starting 7 days after I/RI. In some cases, mice were sacrificed on day 9 after I/RI, and the ischemic kidney was evaluated by flow cytometry for the presence of CD45.2^+^ cells and their F4/80 expression levels. B6.SJL-Ptprca Pepcb/BoyJ naive mice were used as controls for the gating strategy.

### RTEC hypoxia.

RTECs were grown in culture as described above. Glucose- and oxygen-free media were prepared by placing glucose-free DMEM (Gibco, Thermo Fisher Scientific, catalog 11966025) containing 10% FBS, 10 ng/mL EGF, 1% penicillin/streptomycin, and 1% insulin-transferrin-selenium in a hypoxia incubator (O_2_ 20–30 ppm, 37°C) chamber overnight prior to use. On day 7 of RTEC culturing, the cells were washed and placed in the overnight-prepared hypoxic glucose-free media in the hypoxia incubator for 18 hours. This was followed by washing and replacement of the media with glucose-containing media (DMEM/F12, 10% FBS, 1% penicillin/streptomycin) and returning the cells to an incubator with environmental levels of oxygen and 5% CO_2_ for coculturing with BMMΦs.

### RTEC and BMMΦ coculturing.

BMMΦs from *Aif1^+/+^* and *Aif1^–/–^* mice were cultured in M-CSF containing media in Transwell inserts (0.4 μm pore size, catalog 07-200-154, Corning) at 1 × 10^6^ cells per well. M-CSF–containing media were washed prior to coculturing and replaced with DMEM/F12 containing 10% FBS and 1% penicillin/streptomycin. Once the RTECs were retrieved from the hypoxia chamber and washed, Transwell inserts with BMMΦs were placed in the wells with RTECs for 48 hours, after which the RTECs were washed and fixed with 4% paraformaldehyde prior to staining.

### BMMΦ treatment with hypoxia-CM.

*Aif1^+/+^* and *Aif1^–/–^* BMMΦs were generated as described above. RTECs underwent hypoxia followed by reoxygenation, as described above. The resulting culture supernatant (CM) was collected after 48 hours of reoxygenation and centrifuged for 10 minutes at 300*g*, followed by passage through a 0.2 μm filter to remove cell debris. *Aif1^+/+^* and *Aif1^–/–^* BMMΦs (2 × 10^6^) were then incubated with 2 mL CM in 6-well plates for an additional 48 hours, followed by harvesting in TRIzol for reverse transcription quantitative PCR (RT-qPCR).

### Kidney MΦ sorting.

WT and KO mice were subjected to I/RI or sham surgery. Kidneys were harvested, and single-cell suspensions were prepared using mechanical dissociation and collagenase IV digestion. MΦs were then sorted using the EasySep Mouse Biotin Positive Selection Kit II and anti–mouse F4/80 biotin–labeled antibody (STEMCELL Technologies) per the manufacturer’s protocol. The enriched cells were more than 90% pure for F4/80^+^ cells as determined by flow cytometry (data not shown).

### NanoString nCounter.

Kidney MΦs were sorted from ischemic kidneys on day 4 after I/RI as described above. Contralateral nonischemic kidneys were used as controls. RNA was isolated from sorted MΦs using the QIAGEN RNeasy Micro Kit. RNA (10 ng per sample) was used for NanoString hybridization. Gene expression was measured using the nCounter Mouse Myeloid Innate Immunity V2 Panel. Rosalind (version 3.38.01) was used to generate log_2_-normalized counts and expression ratios. The cutoff for DEGs was a fold change (FC) of –1.5 or less or of +1.5 or higher and a *P* value of less than 0.01. Pathway analysis was performed using WikiPathways, and a *P* value of less than 0.05 was considered statistically significant.

### Kidney function assay.

Serum from mice after I/RI was collected at sacrifice. Serum (10 μL) was shipped to the University of Alabama at Birmingham/UCSD (UAB/UCSD) O’Brien Center Core C Resource. Serum creatinine was measured via isotope dilution liquid chromatography tandem mass spectrometry (LC-MS/MS) and reported in mg/dL.

### Flow cytometry and analysis.

Animals were euthanized, and kidneys were perfused with cold PBS and digested with collagenase IV (2 mg/mL for 30 min at 37°C). Cells were stained with fluorochrome-conjugated antibodies for 30 minutes at 4°C. After surface staining, cells were fixed, permeabilized (Cytofix/Cytoperm buffers, BD), and stained for intracellular targets at room temperature (RT) for 1 hour. Cells were acquired on a BD Fortessa and analyzed using FlowJo (version 10.8.1, Tree Star). The following antibodies were used: CD45-BUV395 (30-F11, BD Bioscience), CD11b-BUV805 (M1/70, BD Bioscience), CD11c-PE (M1/70, BioLegend), F4/80-FITC (BM8, BioLegend), Ly6C-eFluor 450 (HK1.6, Invitrogen), I-A/I-E-BV786 (M5/114.15.2, BD Bioscience), Ly6G-APC Cy7 (RB6-8C5, BioLegend), CD206-BV650 (15-2, BD Bioscience), CD86-PE-Cy7 (GL1 RUO, BioLegend), Arg-1–PE (A1exF5, eBioscience). For AIF-1 intracellular staining, purified recombinant anti–Iba1/AIF-1 (EPR16588, Abcam) or isotype rabbit IgG (60024B, R&D Systems) was used for 1 hour at RT, followed by anti–rabbit IgG APC-conjugated secondary antibody (F0111, R&D Systems) for 1 hour at RT. Control staining was performed using kidneys from KO mice. Nonspecific staining was blocked using purified anti–mouse CD16/CD32 (2.4G2, Tonbo Biosciences) and goat serum. Dead cells were excluded using Aqua LIVE/DEAD dye (Molecular Probes). FACS was performed to enumerate the absolute count of a specific cell population per kidney using the following formula: absolute count of a specific cell population per kidney = [(total number of a cell types by FACS/total number of events acquired by FACS)] × [(total number of cells by hemocytometer/weight of kidney used) × total kidney weight].

For generation of the *t*-SNE plot, samples were used from animals sacrificed on the same day, and immunostaining was performed in a single batch to minimize variability. Using the Downsample plugin in FlowJo, the number of collected events was first matched to the same number for each sample and then gated on CD45-expressing live cells. Subsequently, the individual samples were concatenated, and *t*-SNE plot was created using FLOWSOM (84), an R studio plugin for FlowJo, which generated 14 unsupervised cells clusters.

### snRNA-Seq analysis of human kidneys.

A total of 23 snRNA-Seq datasets were selected from publicly accessible datasets (Kidney Precision Medicine Project [KPMP] project; https://www.kpmp.org) (63), Gene Expression Omnibus (GEO) GSE195460 (64), and GEO GSE210622 (65) to generate an integrated human kidney immune cell map (*n* = 8, control healthy kidneys; *n* = 8 AKI; *n* = 7, CKD). First, those samples with very low mitochondrial gene expression, an indicator of efficient stripping of cytoplasm during the isolation of nuclei, were screened and selected. Datasets were then chosen to represent both sexes, all AKI stages, and CKD with fibrosis (high IFTA score) for subsequent analyses (Supplemental Table 1: clinical information). These datasets were further analyzed, as we previously described (85, 86) using the R package Seurat, version 4.2.0, for quality control, dimensionality reduction, and cell clustering (87). The snRNA-Seq matrices were filtered by a custom cutoff (genes expressed in >1 cell, cells expressing >500 genes), and cells with a percentage of mitochondrial genes of less than 5% were included. After removing the potential doublets using DoubletFinder (version 2.04) (88), count matrices from each sample were integrated using Harmony, which corrected potential batch effects (89). To remove any additional confounding source of variation, the mitochondrial mapping percentage was regressed out. A graph-based clustering approach in Seurat was used to cluster the cells in the integrated dataset, and the resolution was set at 1.0. Cluster-defining markers for each cluster were obtained using Seurat’s “FindAllMarkers” command (genes expressed in at least 20% of cells within the cluster; log FC >0.25) with the Wilcoxon rank-sum test. On the basis of the marker genes and manual curation of the gene expression pattern of canonical marker genes in uniform manifold approximation and projection (UMAP) plots, a cell identity was assigned to each cluster. Immune cells expressing *PTPRC* (encoding CD45) were subsequently selected and reclustered for assessment of the *AIF1* gene expression pattern. This approach yielded 9 cell subclusters. Cluster-defining markers for each cluster were again obtained using Seurat’s “FindAllMarkers” command. A cell identity was assigned to each subcluster. Cells that contained mixed marker gene expression or unclassified profiles were then removed to yield the final immune cell map with 5,702 nuclei. To calculate the proportion of *AIF1^+^* cells, those expressing *AIF1* (>0, from the data slot of the sctransform [SCT] assay) were extracted and defined as *AIF1^+^* cells, and other cells within the monocyte and MΦ cultures were defined as negative cells. The number of positive and negative cells was counted, and the percentage of *AIF1^+^* MΦs and monocytes was calculated.

### Statistics.

All data are expressed as the mean ± SEM. Statistical analysis was performed using GraphPad Prism 10.01.02 (GraphPad Software). Normal distribution of the data was assessed using a Q-Q plot generated using GraphPad Prism 10.01.02. Data were analyzed using a 2-sided, unpaired, Student’s *t* test when only 2 groups were compared. When more than 2 datasets were compared, a 1-way or 2-way ANOVA was performed with adjustment for multiple comparisons using Tukey’s test (recommended by Prism). A *P* value of less than 0.05 was considered significant. The only animals excluded from analysis were those that died prior to the planned euthanasia date.

### Study approval.

All animal procedures were approved by the IACUC of Duke University (protocol A260-18-11). For human samples, Duke University IRB approval (protocol pro00108369) was obtained to request previously collected and archived for-cause biopsies of native and transplanted kidneys.

### Data availability statement.

The NanoString Ncounter data supporting the findings of this study are openly available in the Gene Expression Omnibus (GEO) repository (GEO GSE252904). The results here are in whole or part based on data generated by the Kidney Precision Medicine Project (KPMP) (accessed March 11, 2023; https://www.kpmp.org; ref. 63). For snRNA-Seq of human kidney samples, the following additional GEO repositories were utilized GSE195460 and GSE210622. Values for all data points in graphs are reported in the Supporting Data Values file.

## Author contributions

IH and XL designed the study. IH, HS, CZJ, and OF performed the experiments. YC and JP assisted with hypoxia chamber experiments. IH and XL analyzed the data. NRN and SV performed scRNA-Seq analyses of murine samples. HK and TS performed snRNA-Seq analyses of human samples. DNH provided human biopsy samples. IH and XL wrote the manuscript. EBT and XL edited the manuscript.

## Supplementary Material

Supplemental data

Supporting data values

## Version 1 01/21/2025

In-Press Preview

## Version 2 03/03/2025

Electronic publication
