# Supplementary material for: Targeting allograft inflammatory factor 1 reprograms kidney macrophages to enhance repair
Source: J Clin Invest. 2025 Jan 21;135(5):e185146. doi: 10.1172/JCI185146 (PMC11870741; doi:10.1172/JCI185146)
Supplement: Supplemental data [file jci-135-185146-s185.pdf]

Supplementary File

Targeting Allograft Inflammatory Factor-1 reprograms kidney macrophages to enhance repair

Running title: Targeting *Aif1* reprograms macrophages

Irma Husain[1,2], Holly Shah[1], Collin Z. Jordan[1], Naveen R. Natesh[3], Olivia Fay[4], Yanting Chen[5], Jamie Privratsky[5], Hiroki Kitai[1], Tomokazu Souma[1], Shyni Varghese[3,6,7], David N. Howell[4], Edward B. Thorp[8], Xunrong Luo[1,2]

[1]Division of Nephrology, Department of Medicine, Duke University School of Medicine, Durham, North Carolina, USA.

[2]Duke Transplant Center, Duke University School of Medicine, Durham, North Carolina, USA.[3]Department of Biomedical Engineering, Duke University Pratt School of Engineering, Durham, North Carolina, USA

[4]Department of Pathology, Duke University, Durham, North Carolina, USA

[5]Department of Anaesthesiology, Duke University, Durham, North Carolina, USA

[6]Department of Mechanical Engineering and Materials Science, Duke University, Durham, North Carolina, USA

[7]Department of Orthopaedic Surgery, Duke University, Durham, North Carolina, USA

[8]Department of Pathology, Feinberg School of Medicine, Northwestern University, Chicago, Illinois, USA.

SUPPLEMENTARY METHODS

Primary Renal Tubular Epithelial Cell (RTEC) Isolation and Culture

Kidneys were harvested from 6-week-old WT B6 mice in sterile fashion after euthanasia. The kidney tissue was dissociated using a syringe plunger and the fragments obtained were transferred through a stainless-steel sieve (pore size 125 μm) followed by a 100 μm cell strainer (Cat# 431752, Corning). The 100 μm cell strainer was flushed in the reverse direction using PBS into a 50ml conical tube to collect the tubular fragments caught in the strainer. The tubular fragments were then centrifuged for 10 minutes at 300g and resuspended in DMEM/F12 with 2mg/ml of Collagenase IV (Worthington) for digestion at 37°C for 30 minutes. Following digestion, they were resuspended in culture media DMEM/F12, 10% FBS, EGF (10 ng/ml, Cat# SRP3196, Sigma), 1% Penicillin/Streptomycin, 1% Insulin-Transferrin-Selenium (Cat# 41400-045, Gibco) and seeded either into 24 well plates with Poly-L-Lysine (Cat# P4707, Sigma) coated with 12mm coverslips (for co-cultures with BMMΦs) or T75 flasks (for harvesting conditioned media). RTECs were left to grow undisturbed for 4 days, and media changes were performed on day 4 and day 6. On day 7, cells were about 90% confluent and were used for subsequent experiments.

Immunofluorescence Staining and Histopathology

<u>Mouse AIF-1 and F4/80 staining</u>: Frozen kidney sections (5 μm in thickness) were stained using rat anti-mouse F4/80(1:200, A3-1, BioRad) and recombinant rabbit anti-Iba1/AIF-1(1:500, EPR16588, Abcam) overnight at 4°C, followed by secondary staining with goat anti-rat antibody conjugated with DyLight488 (1:500, Novus Biologicals) and goat anti-rabbit IgG(H+L) cross-adsorbed secondary antibody conjugated with AF594 (1:1000, Invitrogen) for 1 hour at RT. Isotype staining was performed with rat IgG2bκ (RTK4530, BioLegend) and rabbit monoclonal IgG (60024B, R&D Systems) antibodies.

<u>Mouse Fibronectin staining</u>: Paraffin embedded formalin fixed sections (5 μm in thickness) were deparaffinized and rehydrated followed by heat-mediated antigen retrieval in a pressure cooker for 15 minutes (buffer EDTA, pH 8.0, ab93680, Abcam). Sections were incubated with rabbit anti-Fibronectin

antibody (1:100, ab2413 Abcam) at 4ºC overnight followed by incubation with goat anti-rabbit IgG H&L (HRP, ab205718, Abcam) at RT for 1 hour. Staining was performed using DAB substrate (Thermo Scientific DAB Substrate Kit, Cat# PI34002) for 10 minutes. Counterstaining with hematoxylin was performed. Isotype control staining was performed using rabbit monoclonal IgG (1:100, 60024B, R&D Systems).

Mouse RTEC TUNEL staining and quantification: Paraffin embedded formalin fixed sections were deparaffinized, rehydrated, treated with Proteinase K (ThermoScientific, 10ug/ml in 10mM Tris/HCl) for 15 minutes at RT, followed by labelling per the In-Situ Cell Death Detection Kit, TMR Red (Sigma Aldrich, Cat# 12156792910) protocol and imaged using a Zeiss Axio fluorescence microscope. Background fluorescence of kidney tissues in the Alexa Fluor 488 channel was used to highlight the kidney architecture. After blinding, cells were quantified (by I.U. and C.Z.J.) by manual counting of TUNEL$^+$ cells per 200x magnification in 10 random non-overlapping fields and mean was calculated and adjusted to the nearest whole number for the final count.

Mouse RTEC Ki67 Staining and Quantification: RTECs growing on coverslips were fixed with 4% paraformaldehyde fixation for 10 minutes at RT. Coverslips were incubated overnight at 4ºC with anti-Ki-67 antibody (0.5 µg/ml, ab15580, Abcam). Secondary antibody incubation was performed using goat anti-rabbit IgG(H+L) cross-adsorbed secondary antibody conjugated with AF594 (1:1000, Invitrogen) at RT for 1 hour. After blinding, cells were quantified (by I.U. and C.Z.J.) by manual counting of both DAPI$^+$ and Ki-67$^+$ cells per 200x magnification in 5 consecutive non-overlapping fields in the centre of the coverslips and mean was calculated and adjusted to the nearest whole number for the final count. Isotype control staining was performed using rabbit monoclonal IgG (0.5 µg/ml, 60024B, R&D Systems). Sections were imaged using a Zeiss Axio fluorescence microscope.

Mouse Kidney Fibrosis and Tubular Atrophy: For evaluation of kidney fibrosis, we deparaffinized and rehydrated 5-µm-thick sections, stained with Picro Sirius Red Stain Kit (ab150681, Abcam) as per manufacturer instructions and stitch images were obtained using Zeiss Axio fluorescence and brightfield

microscope. Tubular atrophy was quantified by blinded histological grading on hematoxylin & eosin stained sections and imaged using Zeiss Axio fluorescence and brightfield microscope.

Immunohistochemical staining of Human Kidney Biopsies: Duke IRB approval (Protocol# Pro00108369) was obtained to request previously collected and archived for-cause biopsies of native and transplant kidneys. Sections were stained with anti-Iba1/AIF-1 antibody [EPR6136(2)] and anti-CD68 antibody by the Duke BioRepository & Precision Pathology Center (BRPC). Images were obtained using Zeiss Axio fluorescence and brightfield microscope.

Image J Analysis for Staining Quantification

Image J based analysis was used to detect the percentage positive areas for both Sirius Red and Fibronectin staining. Blinding was performed prior to analysis. Color images were split into three channels followed by assessment of % red positive area for Sirius Red and % brown positive area for Fibronectin. Thresholds for detection were set based on staining of sham control samples. Same minimum and maximum thresholds were set for all samples stained as a batch. % positive area was quantified for each stitch image of the entire kidney cross-section obtained at 100x magnification.

RNA Extraction and Semiquantitative Polymerase Chain Reaction (PCR)

RNA was extracted from tissue and cells using Trizol Reagent (Invitrogen), followed by cDNA preparation using High-Capacity RNA-to-cDNA Kit (Applied Biosystems). Semiquantitative real-time PCR (ABI Prism 7500) was performed in triplicate using TaqMan master mix. We used the following TaqMan primers/probes: *Col3a1* (Mm00802300_m1), *Chil3* (Mm00657889_mH), *Arg1* (Mm00475988_m1), *Kim-1/Havcr1*(Mm00506686_m1), *Ngal/Lcn2*(Mm01324470_m1), *Il1B*(Mm00434228_m1), *Nos2*(Mm00440502_m1), *Sfrp4*(Mm00840104_m1) and *asma/acta2*(Mm00725412_s1). We used the ddCt method to determine RNA expression, with *Gapdh* (Mm99999915_g1) serving as the internal control. Gene expression was normalized to the expression in sham-operated, WT non-ischemic kidney

samples. For gene expression analysis of BMMΦs after treatment with RTEC-hypoxic-conditioned media, gene expression was normalized to treated *Aif1*$^{+/+}$ BMMΦs.

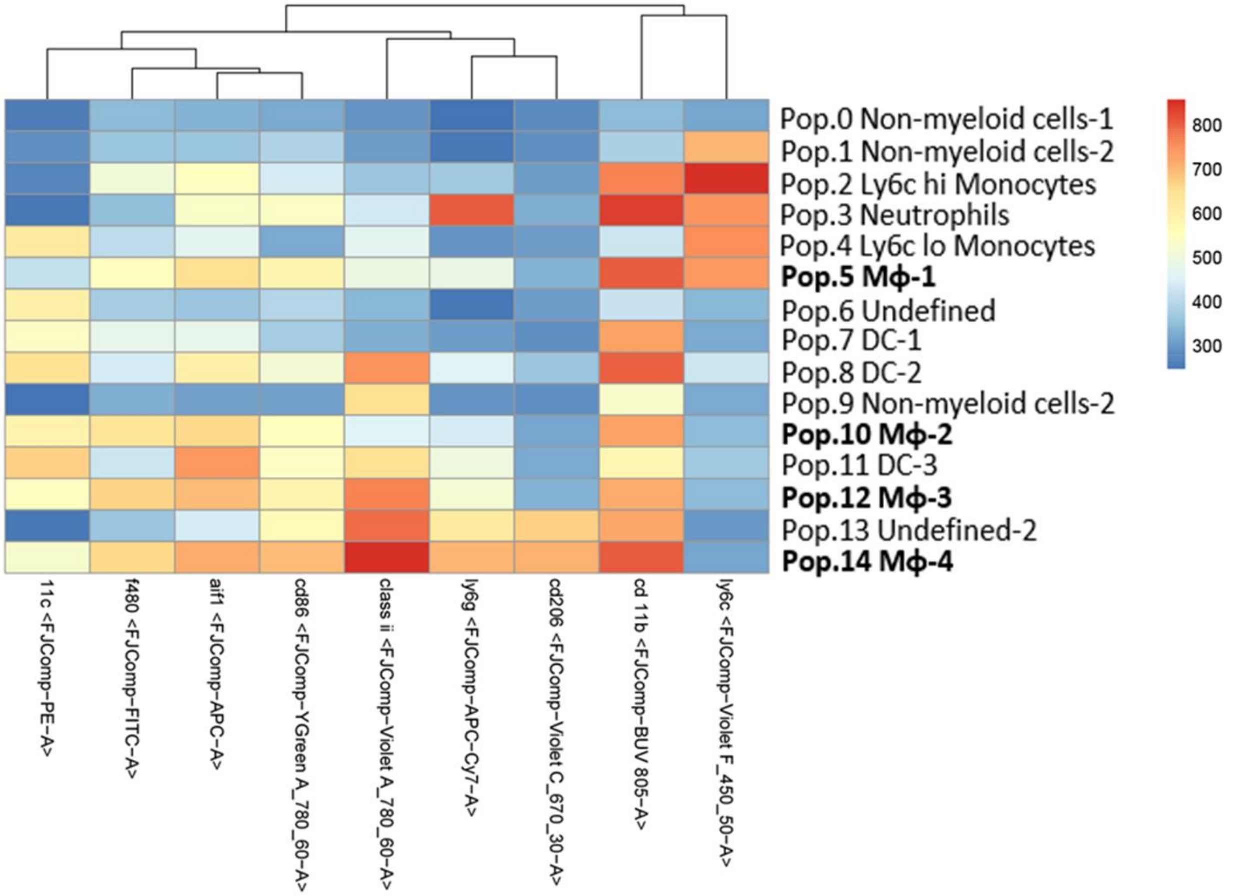

Supplementary Figure 1. Heatmap of selective gene expression of cell clusters (after unsupervised clustering of $CD45^+$ live cells). Cells were combined from sham-operated, day2, day14 and day28 kidneys. n=3 mice per group. MΦ, Macrophage; DC, Dendritic Cell

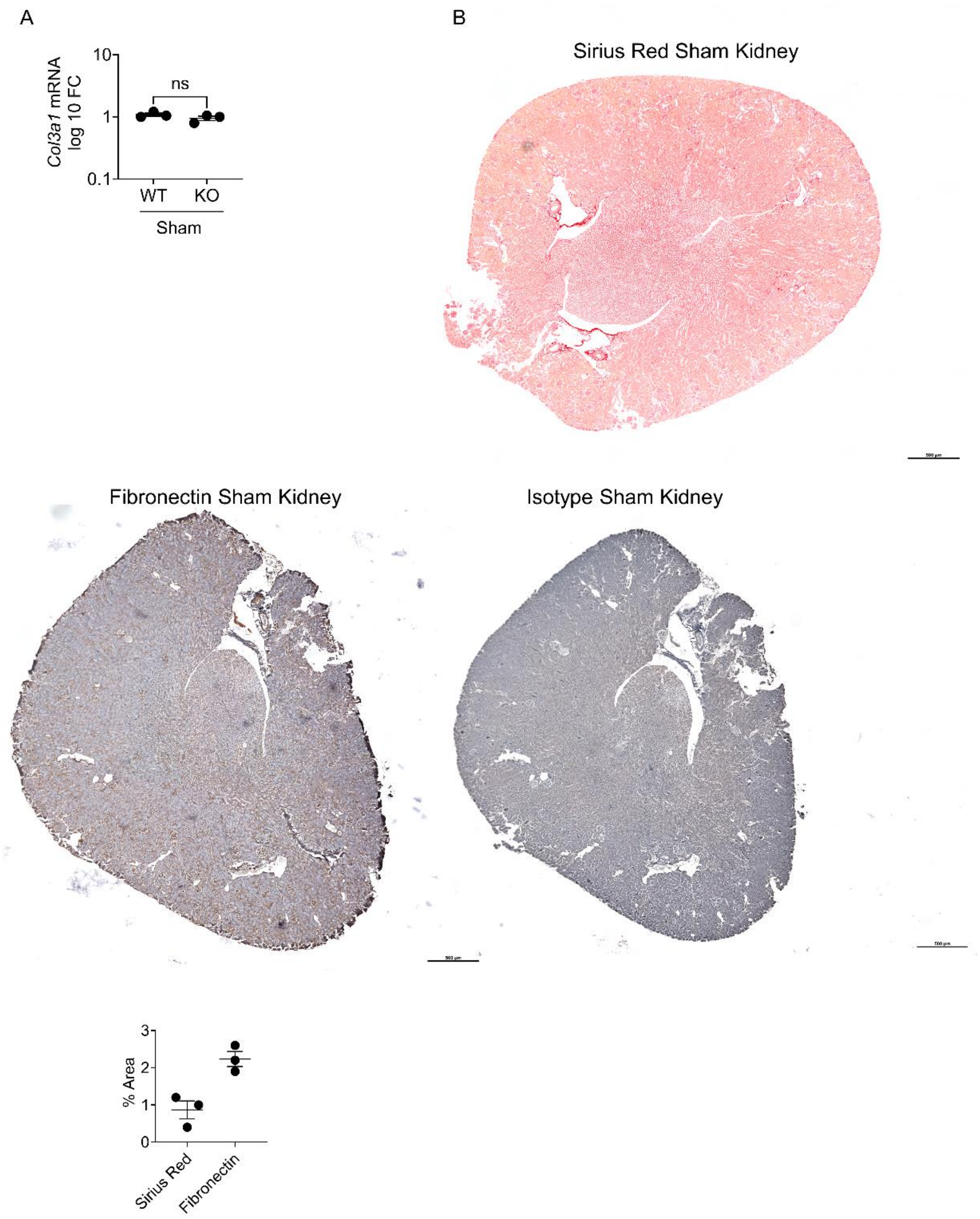

Supplementary Figure 2. Quantification of fibrosis markers for sham-operated mice. (A) Collagen type III alpha chain 1 (Col3a1) mRNA in the indicated groups was measured by reverse transcription quantitative polymerase chain reaction (RT-qPCR). Data is presented on a Log10 axis. (B) Stitch images obtained at 200x magnification for ImageJ quantification of Sirius Red and Fibronectin positive area. Quantification graph shows percentages of kidney area positive for Sirius Red or fibronectin in WT mice. WT, Wild-type; KO, *Aif1* knock-out.

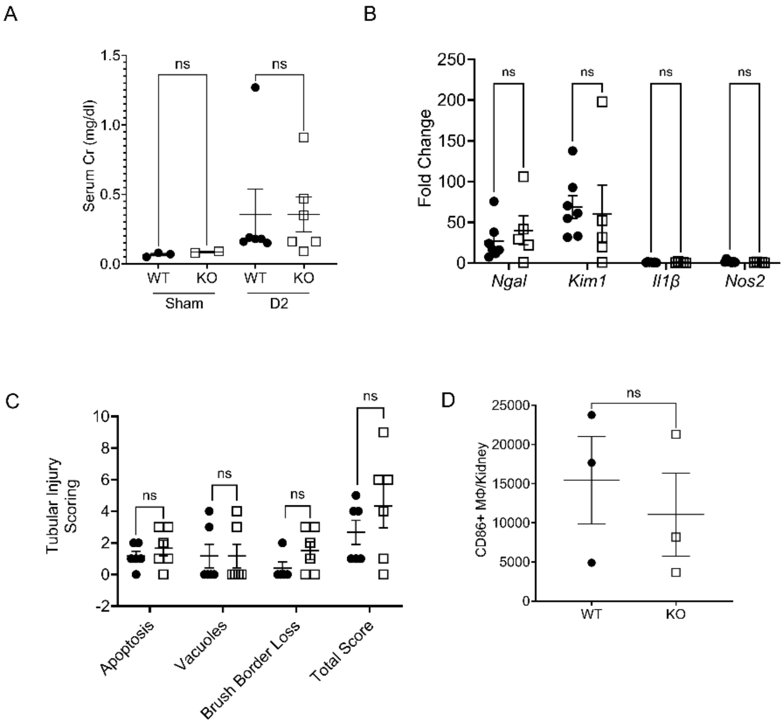

Supplementary Figure 3. Early injury markers in wild-type (WT) and *Aif1* Knock-out (KO) mice are similar. (A) Kidney function measurement by serum creatinine at baseline and at day2 (D2) post I/RI. (B) Tubular injury markers (Neutrophil gelatinase-associated lipocalin (*Ngal*), Kidney injury molecule (*Kim1*), Collagen type III alpha chain 1 (*Col3a1*), and inflammatory markers (Interleukin-1β (*Il1β*) and Nitric oxide synthase-2 (*Nos2*)) mRNA in WT or KO mice was measured by RT-qPCR on day2 post I/RI. Data is represented as fold change normalized to the sham control. (C) Histological scoring of tubular injury on day2 post I/RI performed on H&E and PAS staining. (D) Flow cytometry-based assessment of total number of $CD86^{+}$ macrophages in the kidney on day2 post I/RI. Each symbol represents data from an individual mouse (n=6 each). Error bars are mean ± SEM. ns: not significant. hpf, high power field, WT, wild-type; KO, Aif1 knock-out. For (A-D), each symbol represents data from an individual mouse. Error bars are mean ± SEM. ns: not significant. For (A-C) Statistical analysis was performed using two-way ANOVA test. For (D) Statistical analysis was performed using student’s t test. WT, wild-type; KO, *Aif1* knock-out; MΦ, macrophages.

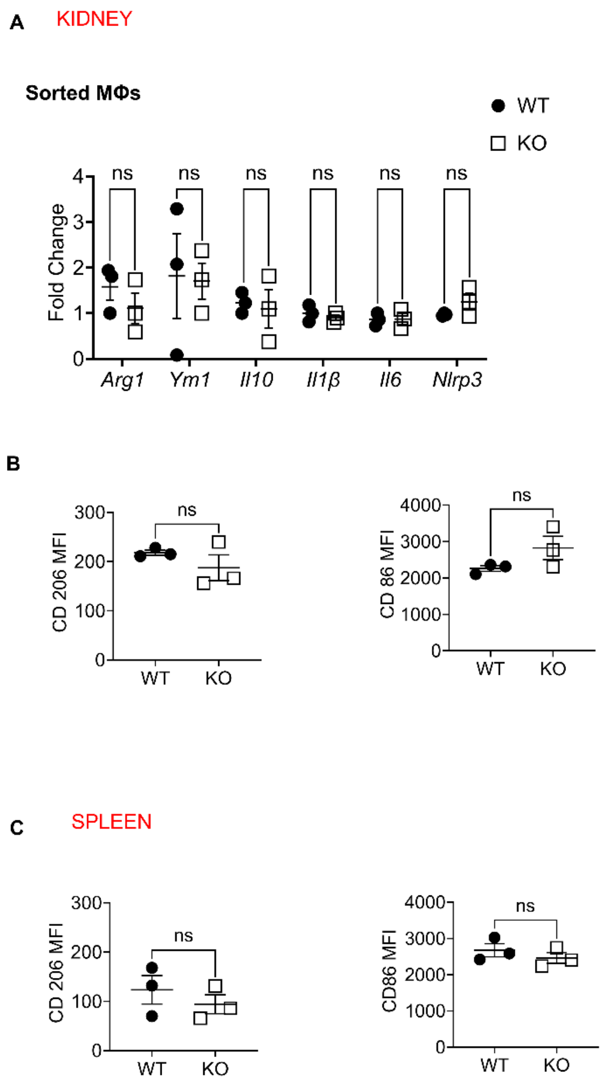

Supplementary Figure 4. Macrophage phenotypes are similar in the kidney and the spleen of unmanipulated WT and KO mice. (A) Macrophages were magnetically sorted from sham operated kidneys from WT and KO mice. Graph shows relative transcripts of reparative markers (Arginase-1*(Arg1), Ym1/Chil3*, Interleukin-10*(Il10)* and inflammatory markers Interleukin-1β *(Il1β), Interleukin-1(Il6)* and *NOD-, LRR- and pyrin domain-containing protein 3(Nlrp3).* (B) Graphs showing kidney macrophage CD206 and CD86 expression by mean fluorescence intensity (MFI). (C) Graphs showing spleen macrophage CD206 and CD86 expression by MFI. Error bars are mean ± SEM. ns: not significant. *$*P<0.05$.* Statistical analysis was performed using student's t test. WT, wild-type; KO, *Aif1* knock-out; MΦ, macrophage.

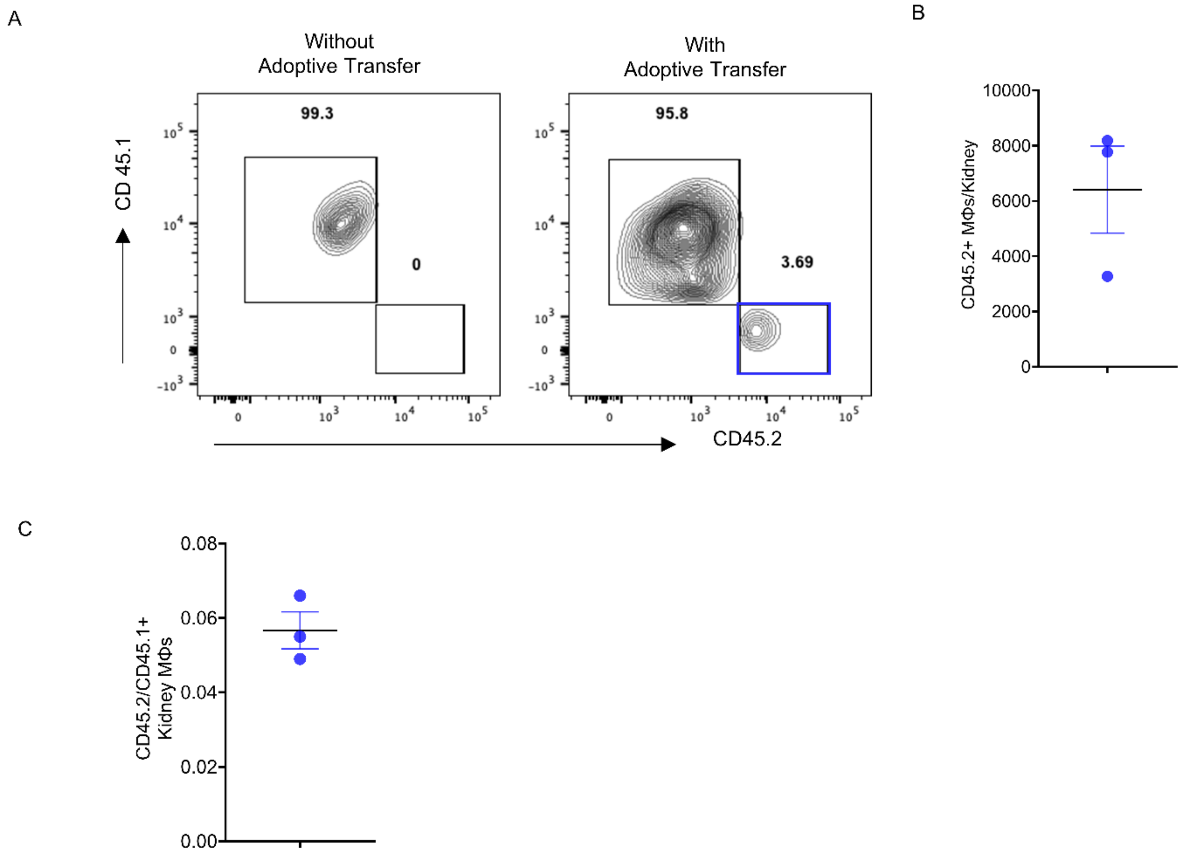

Supplementary Figure 5. Efficacy of bone marrow macrophage adoptive transfer. B6.SJL-Ptprca Pepcb/BoyJ (CD45.1) mice underwent left kidney I/RI and adoptive transfer of 2 x 10^6 BMMΦs (CD45.2) intravenously. Flow cytometry-based assessment of the presence of CD45.1$^+$ and CD45.2$^+$ F4/80$^+$ cells in the ischemic kidney is shown. (A) Contour plots are representative of n=3 mice with adoptive transfers and n=2 without adoptive transfer (naïve controls). (B) Graph showing total CD45.2$^+$F4/80$^+$ cells per kidney. (C) Graph showing the ratio of CD45.2$^+$/CD45.1$^+$ macrophages in the kidney. Each symbol represents an individual mouse. Error bars are mean ± SEM. MΦ, macrophage.

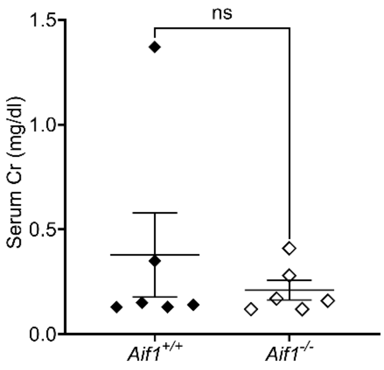

Supplementary Figure 6. Kidney function measurement by serum creatinine (Serum Cr) after adoptive transfer of *Aif1*$^{+/+}$ *or Aif1*$^{-/-}$ BMMΦs. Serum Cr was measured at sacrifice on day28 post I/RI. Each symbol is representative of an individual mice. Error bars are mean ± SEM. ns, not significant. mg/dl, miligrams/deciliter.

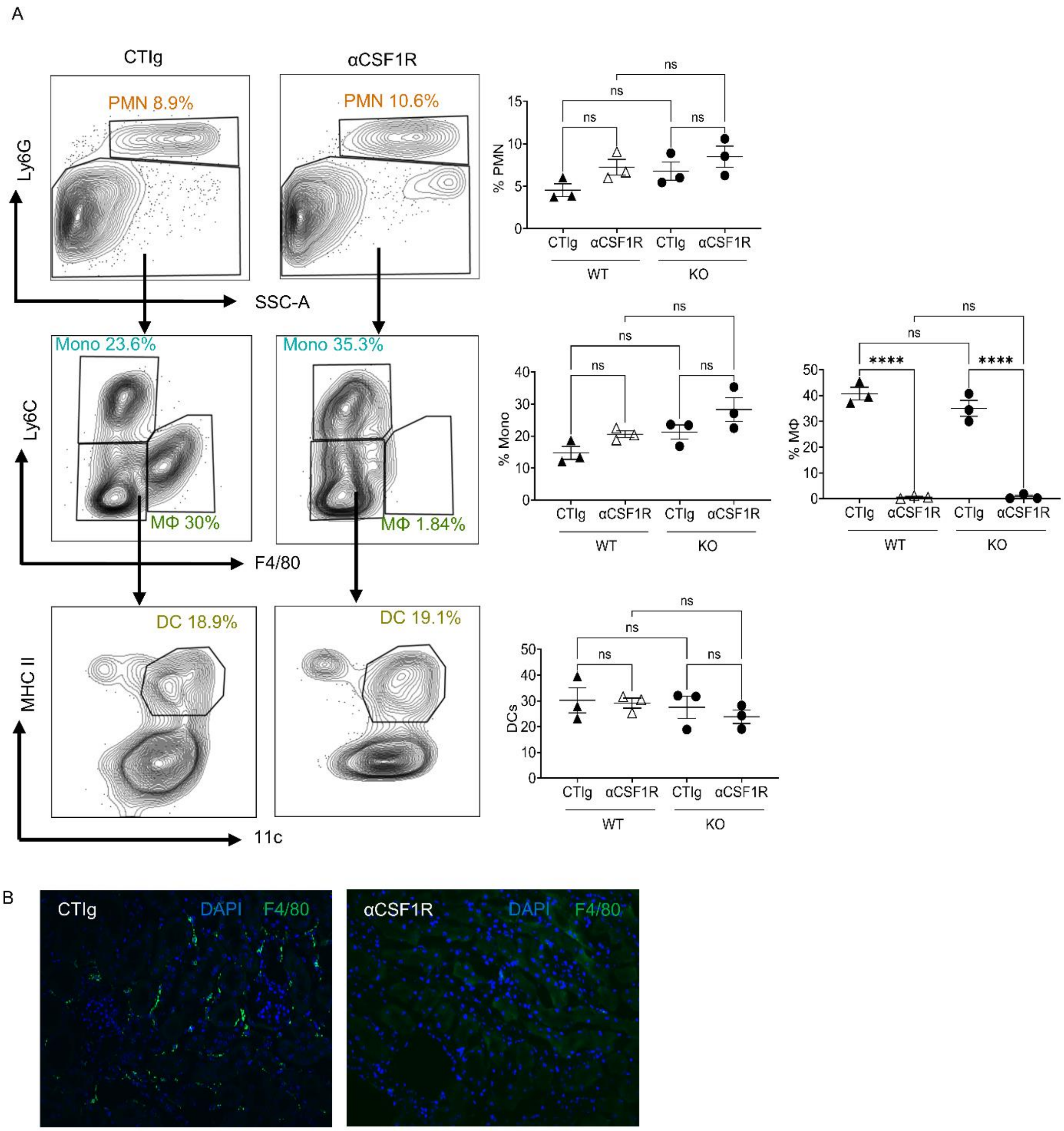

Supplementary Figure 7. Efficacy of kidney MΦ depletion with anti-colony stimulating factor 1R antibody (αCSF1R). Depletion efficacy by aCSF1R versus control immunoglobulin (CTIg) antibody was examined on day 28 post I/RI. (A) Flow cytometry-based assessment of kidney myeloid cells after treatment CTIg (left panels) or αCSF1R (right panels). The graphs show quantification of percentages of each cell type in each group. Each symbol represents data from an individual mouse. (B) Images for immunofluorescence staining for F4/80^{+} MΦs after treatment with CTIg or αCSF1R antibody. Magnification is 100x. Images are representative of n=3 from each treatment group. Error bars are mean ± SEM. ****P<0.001, ns, not significant. PMNs, Neutrophils; MΦ, Macrophage; Mono, Monocyte; DC, Dendritic Cell; WT, wild-type; KO, knock-out.

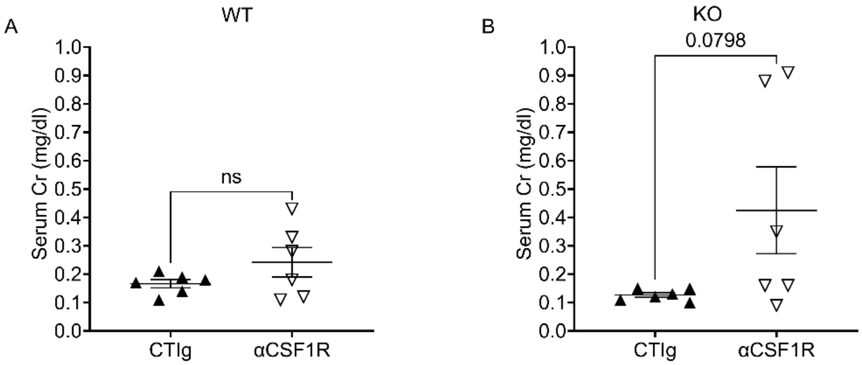

Supplementary Figure 8. Kidney function measurement in the indicated groups at sacrifice on day28 post I/RI. (A) Graph showing comparison of serum creatinine (Serum Cr) in WT mice after treatment with Control Immunoglobulin (CTIg) or anti-CSF-1R antibody (aCSF1R). (B) Graph showing comparison of serum creatinine (Serum Cr) in KO mice after treatment with Control Immunoglobulin (CTIg) and anti-CSF-1R antibody (aCSF1R). Each symbol is representative of an individual mice. Error bars are mean ± SEM. ns, not significant. mg/dl, miligrams/deciliter; WT, wild-type; KO, *Aif1* knock-out.

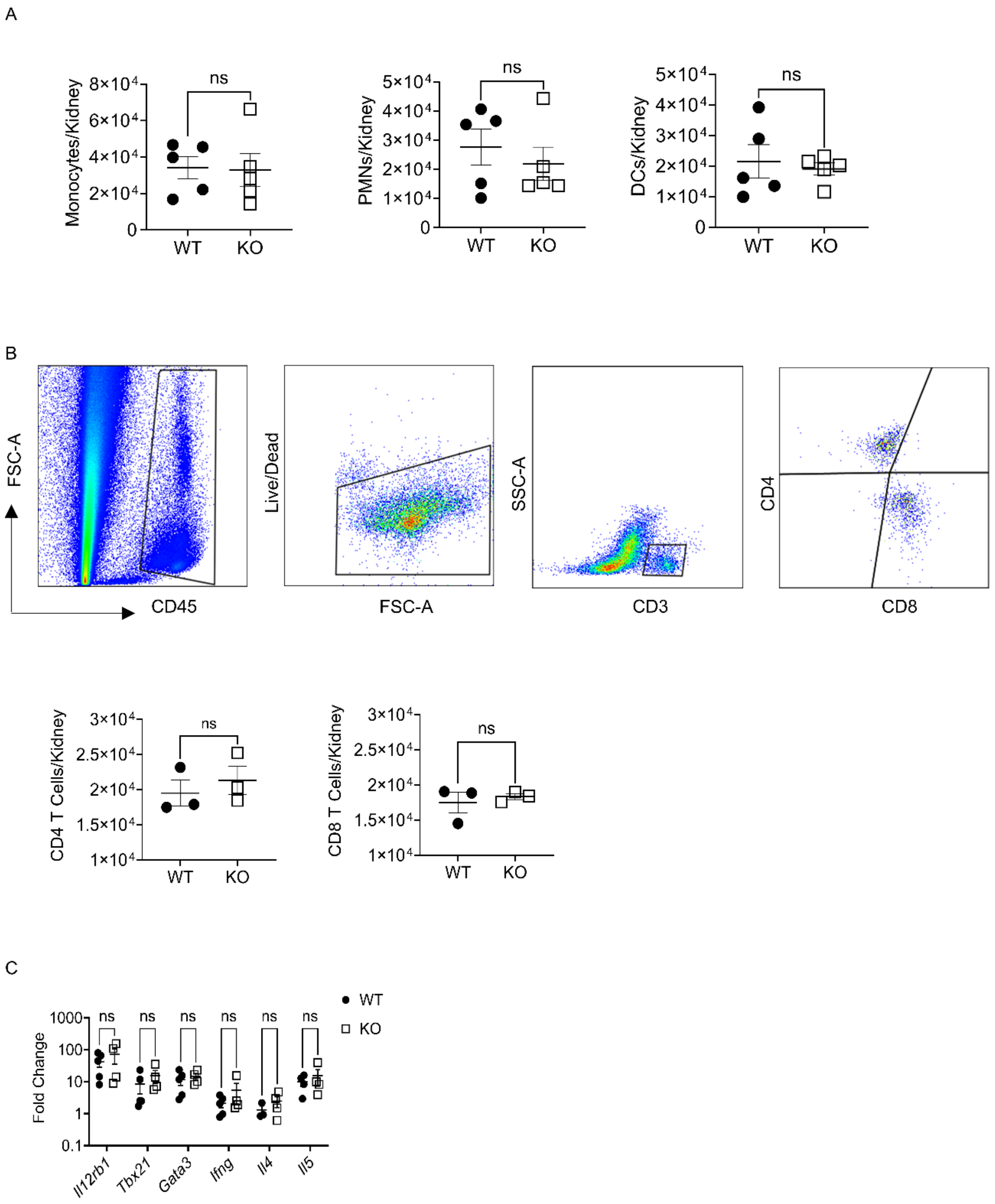

Supplementary Figure 9. Non-macrophage immune cell types in the kidney after I/RI. (A) Graphs showing flow cytometry based enumeration of non-macrophage myeloid cells in the kidneys from day14 post-I/RI WT and KO mice. (B) Gating strategy and flow cytometry based enumeration of CD4 and CD8 T cells in the kidney from day14 post-I/RI WT and KO mice. (C) Gene expression of Interleukin 12 receptor β1 *(Il12rb1),Tbx21, Gata3,* Interferon-gamma (*Ifn-γ),* Interleukin-4 (*Il4)* and Interleukin-5 *(Il5)* by RT-qPCR of kidneys on day14 post I/RI. Data are presented on a log 10 axis. For (C), data was normalized to WT and KO sham surgery mice which was not different at baseline (data not shown). Each symbol represents data from an individual mouse. Error bars are mean ± SEM. ns: not significant. For (A-B) Statistical

analysis was performed using student's t test. For (C) statistical analysis was performed using one way ANOVA. WT, wild-type; KO, *Aif1* knock-out; DCs, dendritic cells; PMNs, Polymorphonuclear cells.

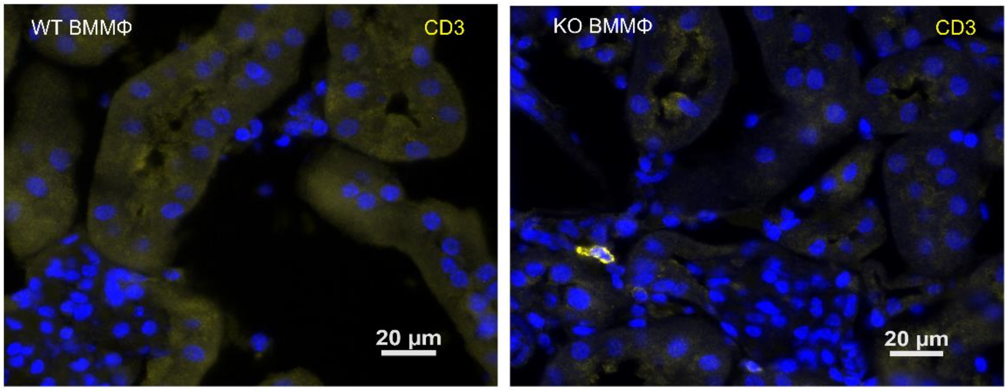

Supplementary Figure 10. Immunofluorescence staining of CD3 on day28 post I/RI in the kidney of WT mice after adoptive transfers with WT or KO BMMΦs. Magnification is 400x. Images are representative of n=6 from each treatment group. WT, wild-type; KO, *Aif1* knock-out.

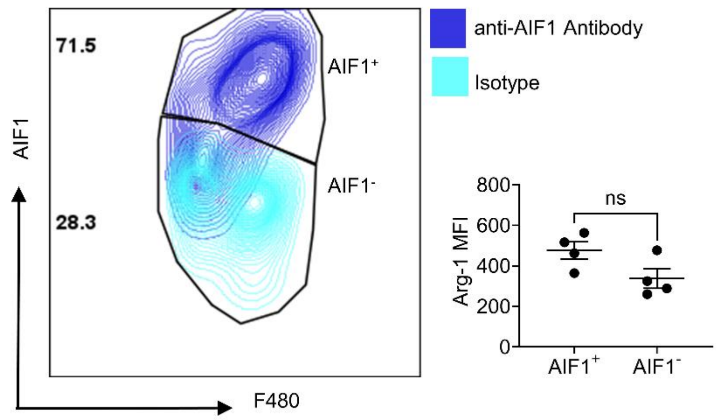

Supplementary Figure 11. AIF-1^{+} and AIF-1^{-} kidney macrophages in WT mice express similar levels of Arginase-1 (Arg-1) on day28 post I/RI. Images are representative of n=4 mice. Graphs show quantification of macrophage arginase-1 in indicated macrophage subsets. Each symbol represents data from an individual mouse (n=4 each). Mean Fluorescence Intensity (MFI) was calculated based on isotype staining. Error bars: mean ± SEM. ns: not significant.

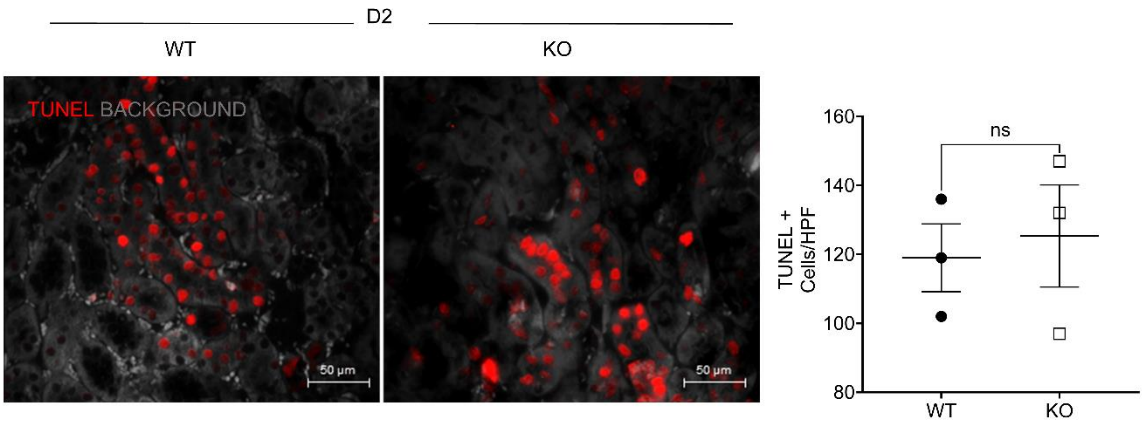

Supplementary Figure 12. TUNEL staining of kidney sections and quantification graph of $TUNEL^{+}$ cells in WT or KO mice on day2 post I/RI. Images are representative of n=3 each in each group. Original magnification: x200. Scale bar = 50 µm. Each symbol represents data from an individual mouse. Error bars are mean ± SEM. ns: not significant. Statistical analysis was performed using student's t-test. WT, wild-type; KO, *Aif1* knock-out; HPF, high power field (200x).

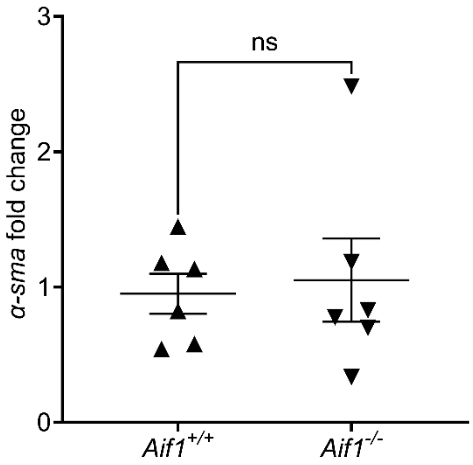

Supplementary Figure 13. Myofibroblast activation measured by alpha smooth muscle actin (*asma)* mRNA. Quantification graph shows fold change of asma mRNA at sacrifice on day28 post I/R after adoptive transfer of *Aif1*$^{+/+}$ *or Aif1*$^{-/-}$ BMMΦs. Each symbol is representative of an individual mouse. Error bars are mean ± SEM. ns, not significant.

A

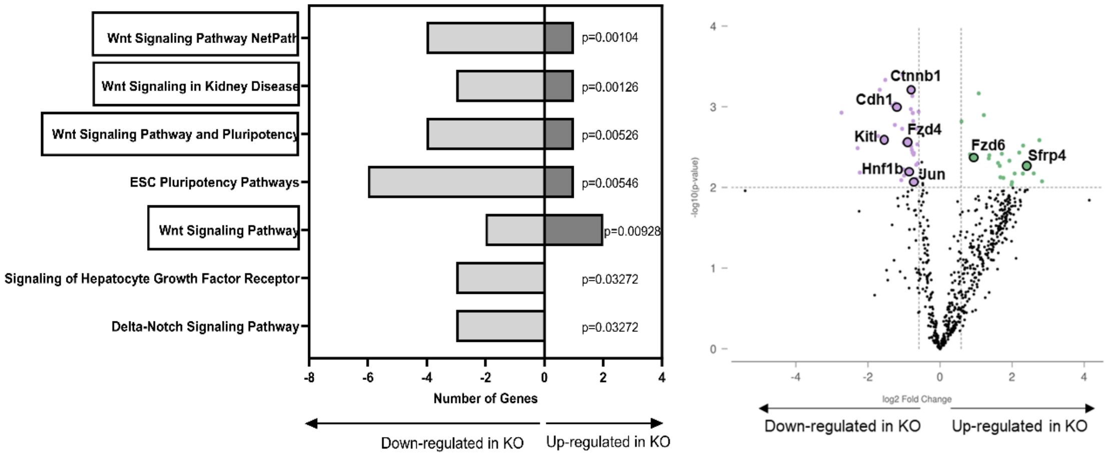

B

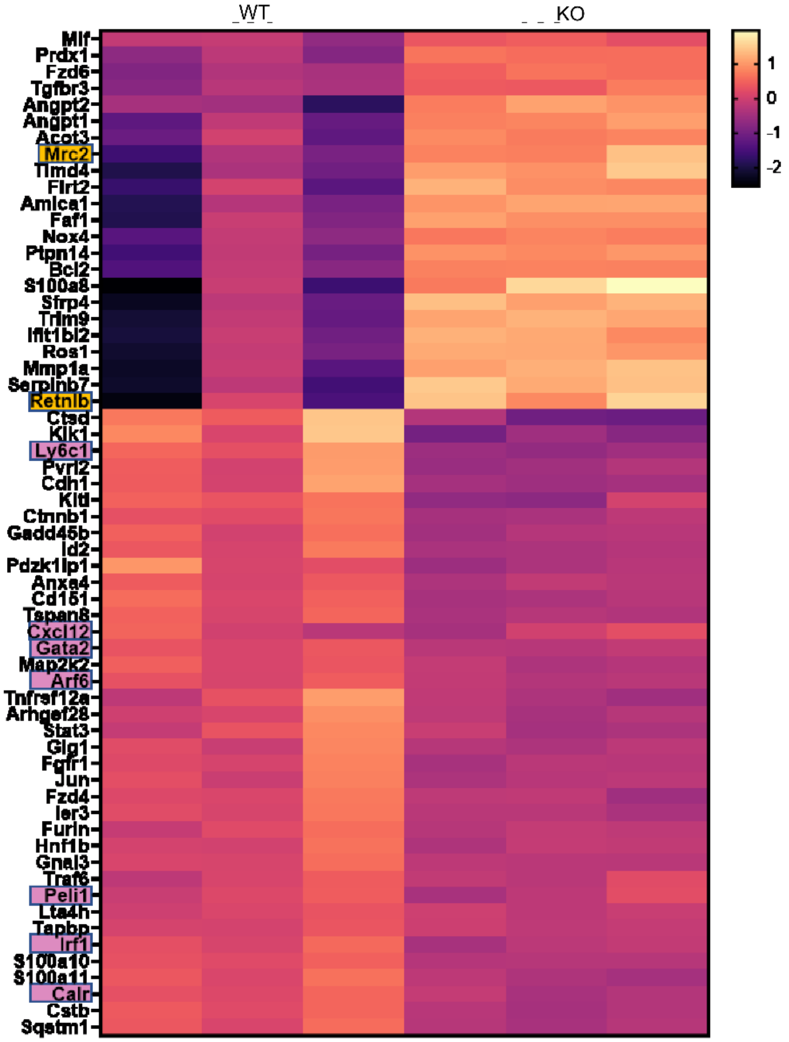

s

Supplementary Figure 14. Nanostring-based differentially expressed genes (DEGs) and pathway analysis comparing *Aif1*$^{-/-}$ (KO) vs. *Aif1*$^{+/+}$ (WT) kidney MΦs post I/RI. Kidney MΦs were

immunomagnetically purified on day4 post I/RI, followed by RNA isolation and Nanostring hybridization. (A) Differentially regulated pathways in *Aif1*$^{-/-/-}$ (KO) vs. *Aif1*$^{+/+}$ (WT) MΦs; x axis represents the number of up-regulated genes (positive value) or downregulated genes (negative value) in KO MΦs; y axis represents the pathway names. The p value for each pathway is labelled adjacent to the pathway bar. Pathways related to Wnt signaling are denoted with a box. Violin plot showing DEGs in the Wnt signaling pathways. Green symbols represent upregulated genes in the KO and purple symbols represent downregulated genes in the KO. Analysis was completed with Rosalind Version 3.38.0.1. (B) Heatmap showing statistically significant DEGs. Genes highlighted in orange are associated with reparative MΦs. Genes highlighted in purple are associated with chronic inflammation and maladaptive repair. Pathway assessment utilized WikiPathways and a gene expression filter of P <0.01 and fold change >=+/-1.5.

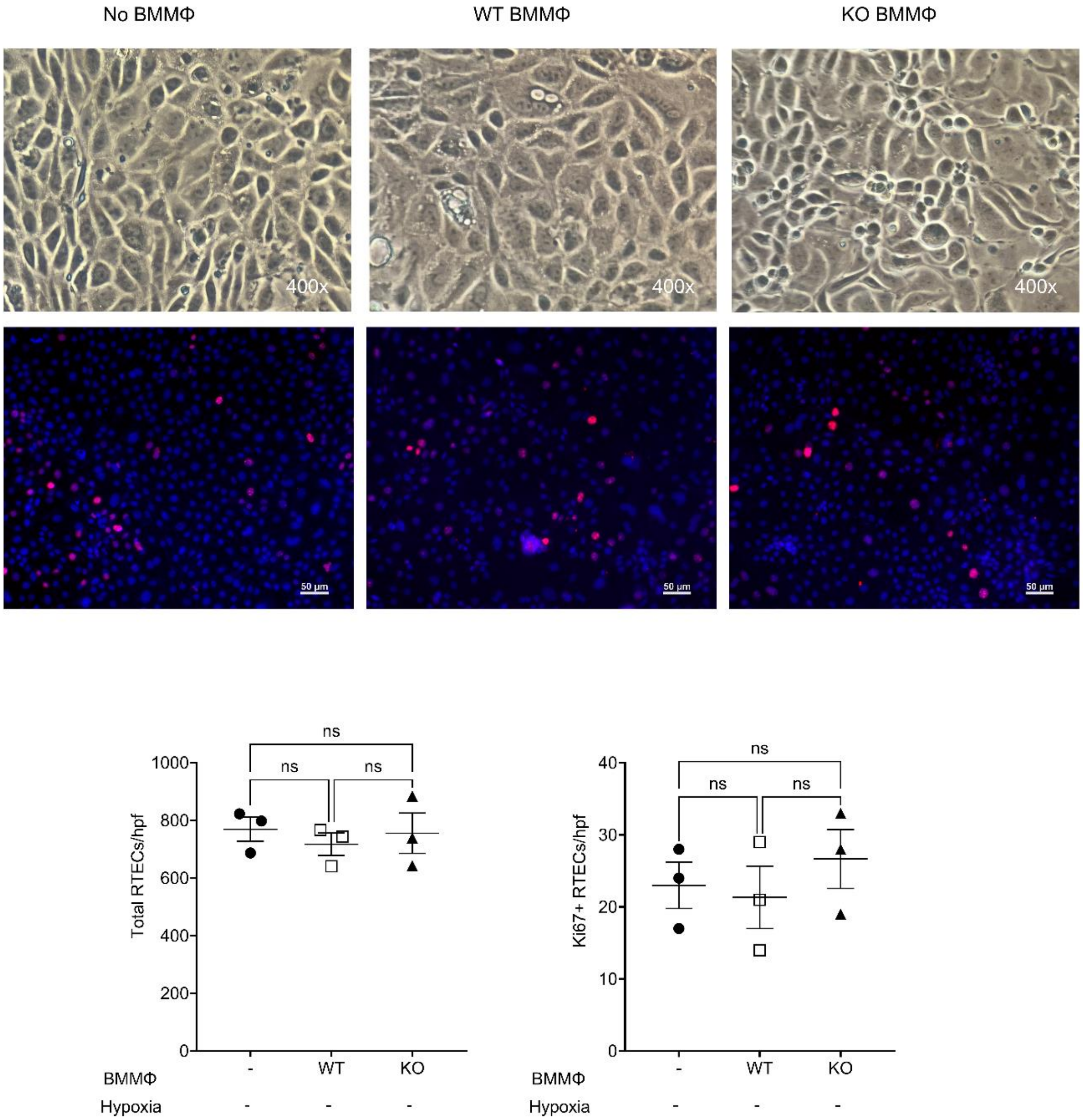

Supplementary Figure 15. WT and KO macrophages co-culturing with RTECs without hypoxia do not alter RTEC viability or proliferation. RTECs were cultured in normal oxygen conditions followed by transwell co-culture with bone marrow macrophages (BMMΦs) from *Aif1* KO and WT mice. Upper panels: representative brightfield microscopy images of RTECs from the indicated groups. Magnification= 400x. Lower panels: representative immunofluorescent image with DAPI (blue) and Ki67(red) staining of RTECs from the same groups. Magnification = 200x. Scale bars = 50 µm. Images are representative of n=3 for each group. Quantification graphs show (1) total RTECs/hpf (DAPI+ cells) and (2) Ki67+ cells/hpf. Each symbol represents data from an individual mouse (n=3 each). Error bars are mean ± SEM. ns: not significant. hpf, high power field, WT, wild-type; KO, Aif1 knock-out.

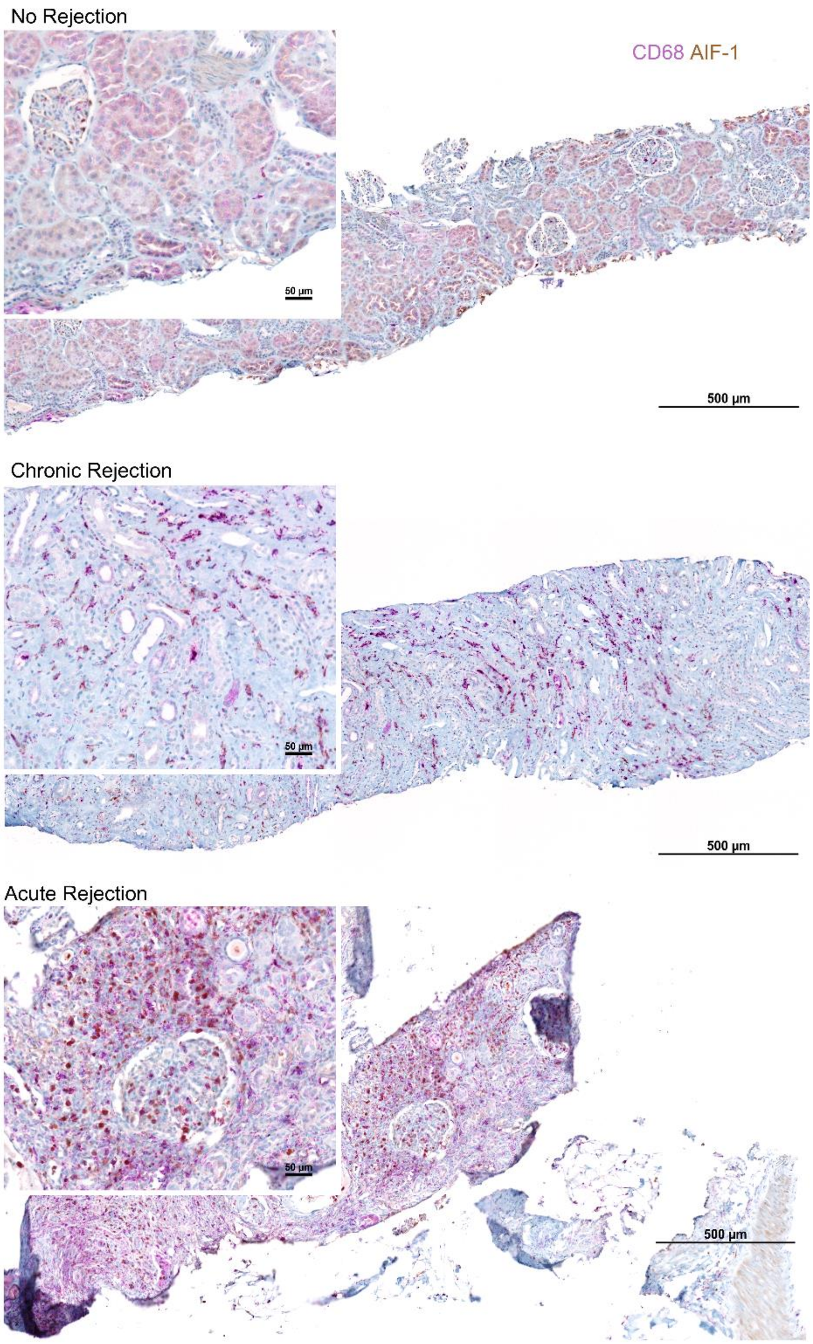

Supplementary Figure 16. Immunohistochemical staining for CD68 (purple) and AIF-1(brown) in human for-cause biopsy samples of post transplant kidneys. Pathological diagnoses are as indicated. Images are representative of n=2 of no rejection, n=2 of chronic rejection and n=3 of acute rejection. Stitch images: 200x magnification, insets: 200x magnification. Scale bars: 500mm and 50mm respectively.

Sample	Dataset	Condition	Sex	Age	AKI stage	eGFR	Creatinine (peak)	IF/TA	Acute tubular injury	Tubular vacuolization
164-1	KPMP	Healthy	male	50-59	N/A	N/A	N/A	N/A	N/A	N/A
164-6	KPMP	Healthy	female	30-39	N/A	N/A	N/A	N/A	N/A	N/A
164-7	KPMP	Healthy	female	40-49	N/A	N/A	N/A	N/A	N/A	N/A
3535_4	KPMP	Healthy	male	NA	N/A	N/A	N/A	N/A	N/A	N/A
KRP461	KPMP	Healthy	male	NA	N/A	N/A	N/A	N/A	N/A	N/A
TN1	Genome_me	Healthy	female	50-59	N/A	N/A	N/A	N/A	N/A	N/A
TN2	Genome_me	Healthy	male	60-69	N/A	N/A	N/A	N/A	N/A	N/A
Cont6	DKD	Healthy	female	59	N/A	100	N/A	1-10%	N/A	N/A
30-10034	KPMP	AKI	female	70-79	stage2	60-69 (base)	N/A	N/A	N/A	N/A
32-10205	KPMP	AKI	male	60-69	stage3	50-59 (base)	N/A	N/A	N/A	N/A
34-10209	KPMP	AKI	male	50-59	stage3	90-99 (base)	N/A	N/A	N/A	N/A
AKI2	Genome_me	AKI	female	60-69	stage3	N/A	1.2-1.4	N/A	1	2
AKI3	Genome_me	AKI	female	50-59	stage3	N/A	3.6-3.8	N/A	1	0
AKI4	Genome_me	AKI	male	50-59	stage3	N/A	1.2-1.4	N/A	1	2
AKI7	Genome_me	AKI	male	70-79	stage3	N/A	2.8-3	N/A	0	0
AKi8	Genome_me	AKI	female	60-69	stage3	N/A	1.8-2	N/A	2	2
29-10012	KPMP	CKD	female	50-59	N/A	30-39	N/A	N/A	N/A	N/A
31-10001	KPMP	CKD	male	70-79	N/A	40-49	N/A	N/A	N/A	N/A
29-10276	KPMP	CKD	female	50-59	N/A	30-39	N/A	N/A	N/A	N/A
31-10000	KPMP	CKD	male	50-59	N/A	20-29	N/A	N/A	N/A	N/A
31-10061	KPMP	CKD	male	60-69	N/A	30-39	N/A	N/A	N/A	N/A
DN4	DKD	CKD	male	78	N/A	73	N/A	26-50%	N/A	N/A
DN5	DKD	CKD	female	51	N/A	101	N/A	26-50%	N/A	N/A

Supplementary Table 1**.** Clinical characteristics of patient samples and their respective data origins included in single nuclear sequencing re-analysis of human native kidney biopsies in Figure 8. KPMP, kidney precision medicine project; DKD, diabetic kidney disease; Genome_me, Genome Medicine; N/A, not available; IF/TA, interstitial fibrosis tubular atrophy; eGFR, estimated glomerular filtration rate.
